# Integrated Multi-Omics Analysis Reveals Stage-Specific Molecular Modules Regulating Uterine Function and Fecundity in Large White Pigs Across Reproductive Lifespan

**DOI:** 10.3390/biology14111589

**Published:** 2025-11-13

**Authors:** Wenwu Chen, Fang Yang, Jingwen Liu, Lei Yi, Sui Liufu, Kaiming Wang, Yan Gong, Zhi Li, Haiming Ma

**Affiliations:** 1 College of Animal Science and Technology, Hunan Agricultural University, Changsha 410128, China; cww1242646778@163.com (W.C.); y621829@163.com (F.Y.); 15093946542@163.com (J.L.); ylasoi@163.com (L.Y.); liufusui0816@163.com (S.L.); m15116529648@163.com (K.W.); 13910008175@139.com (Y.G.); 2 Key Laboratory of Livestock and Poultry Resources (Pig) Evaluation and Utilization, Ministry of Agriculture and Rural Affairs, Changsha 410128, China; 3 Yuelushan Laboratory, Changsha 410128, China; 4 Hunan Key Laboratory for Conservation and Utilization of Biological Resources in the Nanyue Mountainous Region, Hengyang Normal University, Hengyang 421008, China; zhili963@foxmail.com

**Keywords:** white large pigs, uterine, multi-omics, reproductive, fecundity

## Abstract

This study systematically explored the regulatory mechanisms of uterine function across four reproductive stages: sexual maturity sow (SMS), low-yield sow (LYS), high-yield sow (HYS), and culled sow (CS) in Large White (LW) pigs through integrated transcriptomic, proteomic, and metabolomic analyses. Twelve healthy LW sows were selected, and uterine tissues were collected for multi-omics detection. Combined with bioinformatics analysis, molecular regulatory networks were constructed. Results showed that transcriptomics identified 12 types of alternative splicing and 1243 novel genes, which were enriched in energy metabolism and signal transduction pathways. Proteomics revealed 430 differentially co-expressed proteins, indicating high protein synthesis activity in the SMS stage and extracellular inflammatory characteristics in the CS stage. Metabolomics detected numerous differential metabolites, among which XTP and DHA ethyl ester were associated with high fecundity and aging, respectively. Integrated multi-omics analysis identified hub genes such as PLA2G4A, which influence reproductive performance by regulating inflammatory and metabolic balance, and clarified stage-specific “gene–protein–metabolite” modules. This study provides a molecular map for understanding dynamic changes in uterine function in Large White pigs and offers a theoretical basis for optimizing reproductive lifespan and breeding strategies.

## Simple Summary

Pigs are important for farming, and a key part of their ability to produce healthy piglets lies in their uterus. However, we do not fully understand how the uterus works as pigs go through different stages of their breeding life—from when they first mature, to when they produce few piglets, many piglets, and finally when they stop breeding. This study aimed to figure out these changes by looking at genes, proteins, and small molecules in the uterus of Large White pigs across these four stages. We found new genes and proteins that change with each stage: young, mature sows have high protein production, while culled sows show signs of inflammation. We also found molecules linked to more piglets (like Xanthosine 5′-triphosphate, XTP) and aging (like a form of Docosahexaenoic Acid, DHA). These findings show how the uterus changes at each stage, helping farmers improve how they breed and care for pigs, which can boost farm efficiency and food production.

## 1. Introduction

The uterus is the core organ governing pregnancy success and reproductive lifespan in sows, directly determining litter size, piglet survival rate, and culling rate—key factors that underpin the economic efficiency of global commercial swine production [1]. Among dominant commercial pig breeds, LW pigs are the most widely used maternal line in intensive farming systems (e.g., the three-way cross ‘Duroc × Landrace × LW pigs’). They account for a significant majority of sows in commercial herds (estimated at over 65% [2]) due to their superior fecundity, maternal ability, and adaptability to large-scale production [3]. Despite this, the integrated molecular networks (transcriptomic, proteomic, metabolomic) regulating uterine development, peak reproductive function, and age-related decline in LW pigs remain poorly characterized—creating a vital gap between basic research and practical breeding/management strategies for this key maternal breed.

Multi-omics technologies have emerged as powerful tools to address this gap, as they enable multi-dimensional dissection of tissue physiology by linking “gene expression–protein synthesis–metabolite accumulation,” thereby avoiding the limitations of single-omics analyses. For instance, Huang et al. compared the endometrial transcriptomes of Meishan pigs (a Chinese indigenous breed with high fecundity) and LW pigs during early (day 15) and mid-gestation (days 26 and 50), revealing that differentially expressed genes (DEGs) in LW endometrium were primarily enriched in steroid metabolism and retinoic acid receptor signaling pathways—two cascades critical for mediating estrogen/progesterone responses and endometrial receptivity, which are essential for maintaining the high parity performance of LW sows [4]. Complementing this transcriptomic data, their metabolomic analysis further showed that antioxidant metabolites (e.g., glutathione) and energy metabolites (e.g., pyruvate) were significantly elevated in LW uterine fluid during mid-gestation, a pattern that aligned with the high energy and redox homeostasis requirements of rapidly developing embryos—providing initial evidence for breed-specific metabolic adaptation in LW pigs’ uterine microenvironment.

Beyond early pregnancy, proteomic studies have uncovered key regulators of embryo implantation in porcine uterine fluid, with implications for LW pigs’ reproductive efficiency. During the embryo implantation window (day 12 of pregnancy), S100A8 protein—isolated from porcine uterine fluid exosomes—promotes the proliferation and migration of embryonic trophoblast cells by activating the Wnt/β-catenin pathway [5]; notably, its expression level is positively correlated with lactate content in uterine fluid, suggesting a coordinated role of protein signaling and energy metabolism in supporting implantation [6]. For LW pigs specifically, this mechanism may be particularly relevant, as their high litter size relies on efficient multi-embryo implantation—a process that depends on stable trophoblast–uterine crosstalk. Additionally, mid-gestation endometrium of LW sows exhibits high expression of vascular endothelial growth factor D (VEGFD) and collagen α4 chain (COL4), which are tightly associated with uterine angiogenesis and stromal remodeling [7]; these processes are critical for establishing functional maternal–fetal blood exchange, which is a key determinant of fetal survival in high-fecundity LW litters.

Comparative studies across breeds have further highlighted the need for targeted research in LW pigs. For example, during mid-gestation (days 49 and 72), the endometrium of Meishan pigs exhibits significantly higher expression of extracellular matrix (ECM) remodeling proteins (e.g., MMP2, TIMP3) and antioxidant enzymes (e.g., SOD1) than Duroc pigs (a breed with relatively lower fecundity), with high expression of these proteins positively correlated with embryo survival rate [8]. Transcriptomic analysis further confirmed that the expression of Hoxa10—a master gene regulating endometrial receptivity—was 2.3-fold higher in Meishan than in Duroc endometrium [9]. However, how these ECM and antioxidant pathways are regulated in LW pigs (which balance high fecundity with commercial growth traits) remains unclear. Metabolomically, uterine fluid from high-yielding sows (including LW lines) contains higher levels of essential amino acids (e.g., arginine, leucine) [10] and polyunsaturated fatty acids (e.g., DHA) compared to low-yielding counterparts; these metabolites enhance implantation success by promoting embryonic cell proliferation and suppressing excessive inflammatory responses [11]. Yet, the specific “transcript–protein–metabolite” modules driving these differences in LW pigs—especially during their peak reproductive stage (parity 4) and pre-culling decline—have not been systematically mapped.

To fill these gaps, the present study aimed to (1) perform integrated transcriptomic, proteomic, and metabolomic analysis of uterine tissues from LW pigs at three key developmental stages: sexual maturity (6 months, pre-first parity), peak reproduction (parity 4), and pre-culling (parity 9, with confirmed reproductive decline); (2) identify stage-specific molecular modules (genes, proteins, metabolites) regulating uterine function (e.g., endometrial receptivity, nutrient transport, inflammatory homeostasis) unique to LW pigs; and (3) validate key molecular markers associated with sustained fecundity in LW sows, providing a theoretical basis for optimizing their reproductive lifespan in commercial production.

## 2. Materials and Methods

### 2.1. Animals and Sampling

A total of 12 healthy LW sows were selected from a commercial intensive pig farm ([Xinwufeng Co., Ltd., located in Changde, China]) with uniform feeding and management conditions (ad libitum access to a standard commercial gestation diet meeting the nutrient requirements of NRC (2012) for sows, and free access to clean drinking water, Supplementary Materials S1). The sows were divided into 4 groups: sexual maturity sows (SMS), low-yield sows (LYS), high-yield sows (HYS), and culled sows (CS) (*n* = 3 per group) based on their reproductive status and performance (the average number of piglets born in the LYS group of 4 pregnancies was 9, while that in the HYS group of 4 pregnancies was 14; CS comprised sows that had produced 9 litters). Tissue samples were collected from the right uterine horn of each sow. To minimize the physiological variability associated with different estrous stages in uterine tissues, all samples in this study were collected from sows on day 10 of the estrous cycle. Specifically, four samples were allocated for transcriptomic analysis, three for proteomic analysis, and six for metabolomic analysis. All samples were collected within 30 min post-slaughter, immediately frozen in liquid nitrogen, and subsequently stored at −80 °C until further use. All animal-related procedures were conducted in accordance with the guidelines of the Institutional Animal Care and Use Committee of Hunan Agricultural University (No. 2021-13).

### 2.2. RNA Library Preparation and Transcriptome Sequencing

Total RNA was extracted using TRIzol reagent (Life Technologies, Carlsbad, CA, USA), with integrity verified by Agilent 2100 Bioanalyzer (RIN ≥ 8.0) (Agilent Technologies, Santa Clara, CA, USA)). Libraries were constructed from 1 μg RNA using the Hieff NGS Ultima Dual-mode Kit (Yeasen, Shanghai, China) with unique barcodes, followed by paired-end sequencing (150 bp) on an Illumina NovaSeq 6000 platform.

Raw data were filtered (adapter removal, low-quality reads discarded) via BMK-Cloud. Clean reads were aligned to the Sus scrofa 11.1 genome using HISAT2 (≤1 mismatch) and assembled with StringTie. Functional annotation was performed against NCBI nr, Pfam, KOG/COG, Swiss-Prot, KEGG, and GO databases.

Transcript abundance was quantified as FPKM. DEGs were conducted using DESeq2 (version 1.40.0) with the thresholds adjusted *p* < 0.01 and |log_2_FC| ≥ 1. Enrichment analysis was performed via clusterProfiler, and protein interaction networks were visualized using STRING (version 11.5) and Cytoscape. The raw transcriptome data were filtered by BMK-Cloud, and the sequencing batch effect was corrected using the SVA package (R v4.2.1).

### 2.3. Proteome Detection and Analysis

Proteins were extracted in RIPA buffer (with protease inhibitors), ultrasonicated (300 W, 3 s on/3 s off for 10 min), and centrifuged (12,000× *g*, 4 °C, 10 min). Supernatants were reduced with 10 mM DTT, alkylated with 55 mM IAM, and precipitated with acetone. Pellets were digested with trypsin (1:50, 37 °C overnight), desalted, and analyzed by LC-MS/MS on an Orbitrap system (C18 column, 3 μm, 100 × 180 mm; gradient elution with 0.1% formic acid/acetonitrile).

The FDR for all protein identification and differential analysis was strictly controlled at ≤1%. This standard was achieved through the “target-decoy database search strategy” of Spectronaut 14.2 software. The decoy database is a randomly shuffled Sus scrofa protein sequence library, which ensures that the identified differentially accumulated proteins (DAPs) are free from false-positive interference.

The label-free quantification method was adopted, which was specifically implemented through the “MS1 intensity-based quantification” module of Spectronaut 14.2 software. The method uses the MS1 intensity of peptides as the basis for quantification, which was then used for the subsequent screening of DAPs (original screening criteria: |log_2_FC| ≥ 0.26 and *p* < 0.05). Multivariate analysis (PCA, K-means clustering) and functional annotation (COG/GO/KEGG) were performed.

### 2.4. LCMS Non-Targeted Metabolomics Analysis

Metabolites were analyzed following the below detailed procedures: First, approximately 50 mg of uterine tissue was taken, and 500 μL of pre-cooled methanol–water mixture (volume ratio 8:2) was added. After vortex oscillation for 30 s, ultrasonic extraction was performed under ice bath conditions for 20 min (power 300 W, 3 s on/3 s off). Then, centrifugation was conducted at 12,000× *g* for 15 min at 4 °C; the supernatant was transferred to a new centrifuge tube, an equal volume of acetonitrile was added, and after vortexing, centrifugation was performed again at 10,000× *g* for 10 min at 4 °C to collect the supernatant. The supernatant was freeze-dried under vacuum, then reconstituted with 100 μL of mobile phase (0.1% formic acid water–acetonitrile, volume ratio 9:1), filtered through a 0.22 μm organic phase filter membrane, and transferred to a sample vial. During sample pretreatment, L-2-chlorophenylalanine (purchased from Sigma-Aldrich, concentration 2 μg/mL) was used as the internal standard: 10 μL of internal standard solution was added to 100 μL of homogenized uterine tissue sample to correct for metabolite loss during sample pretreatment.

Subsequently, metabolites were detected using a Waters Acquity UPLC system coupled with a Xevo G2-XS QToF mass spectrometer (HSS T3 column, 1.8 μm, 2.1 × 100 mm; gradient elution with 0.1% formic acid/water and acetonitrile). For detection quality control: 3 injections of blank solvent (0.1% formic acid water-acetonitrile) were run before each batch of samples to clean the system; during detection, 1 quality control (QC) sample containing the internal standard was inserted every 10 samples, and the detection was continued only when the coefficient of variation (CV) of the internal standard peak area was <10% and the aggregation degree of QC samples in principal component analysis (PCA) was ≥95%. MS was operated in MSe mode with alternating collision energies (0 V and 10–40 V).

Raw data were processed via Progenesis QI (version 4.0) (peak picking, alignment, normalization). Compounds were identified against METLIN and a custom library (mass error <5 ppm). Multivariate analysis (PCA, OPLS-DA with 200 permutations) was performed. Differential metabolites were selected by |log_2_FC| ≥ 0.3, *p* < 0.05, and VIP > 1, followed by KEGG/HMDB/LipidMaps pathway enrichment. Metabolome data were analyzed using the ‘batch correction’ function of Progenesis QI to eliminate deviations among different detection batches.

### 2.5. Multi-Omics Integrated Analysis

To explore associations among transcriptomic, proteomic, and metabolomic datasets, regulatory network analysis was performed for four pairwise comparison groups (SMS vs. LYS, LYS vs. CS, SMS vs. CS, LYS vs. HYS). For each group, previously identified DEGs (adjusted *p* < 0.01, |log_2_FC| ≥ 1), DAPs (|log_2_FC| ≥ 0.26, *p* < 0.05), and differentially expressed metabolites (DEMs) (|log_2_FC| ≥ 0.3, *p* < 0.05, VIP > 1) were integrated as core molecular entities. Networks were constructed by mapping direct or indirect associations between these molecules. Based on the STRING database (confidence score > 0.7), the known molecular interaction relationships were screened. Combined with the molecules co-enriched by the KEGG pathway (*p* < 0.05), the regulatory network was constructed using Cytoscape 3.9.

To investigate the relationship between DEGs and DAPs across all four groups, restricted correspondence analysis was conducted. For each pairwise comparison group, the top 50 DEGs (ranked by adjusted *p*-value) were selected, and their corresponding associations with DAPs were analyzed based on (1) shared involvement in uterine growth and development-related pathways; (2) significant correlation in expression trends (Pearson correlation coefficient > 0.6). Corresponding DAPs were matched to these DEGs.

Spearman correlation analysis was performed to evaluate the strength of associations between DEGs and DEMs in each pairwise comparison group. Correlation coefficients were calculated using R software (v4.2.1), with absolute correlation coefficient > 0.7 considered a strong association. Genes with strong correlations to multiple metabolites were identified as “hub genes,” and their positional roles in the gene–metabolite interaction network were determined.

### 2.6. Real-Time PCR Quantification of mRNAs

The sequencing data were validated by qPCR. Primer sequences were designed using the NCBI database and synthesized by Tsingke Biotechnology (Beijing, China). All primer sequence information is provided in Table 1. GAPDH was used as a reference gene, and relative gene expression levels were calculated using the 2^−ΔΔCt^ method.

## 3. Results

### 3.1. Analysis of Transcriptome Sequencing Data

**Figure 1:**
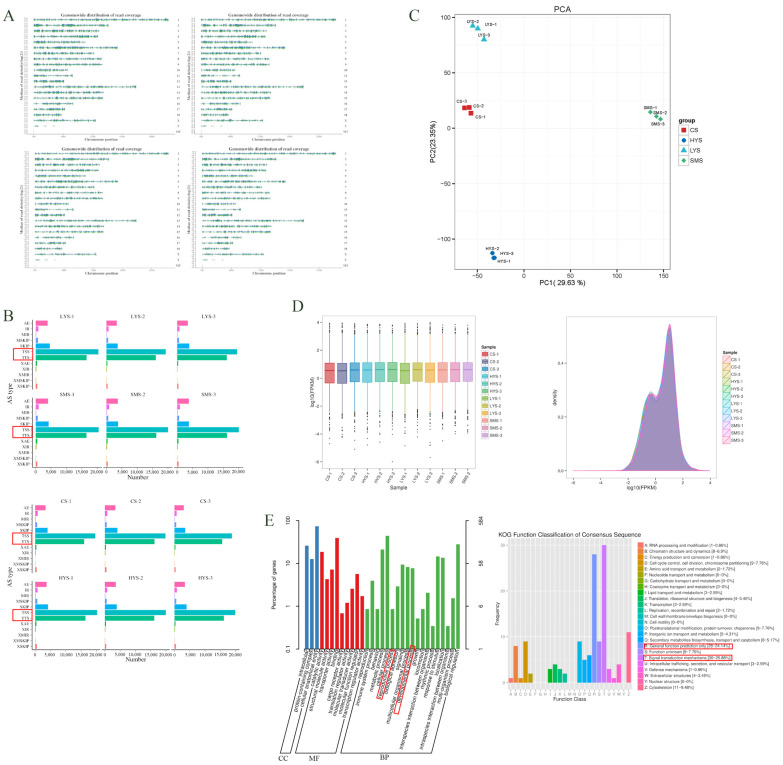
Genome coverage of the porcine transcriptome sequencing data: Sample groups are positioned in the figure as follows: SMS (upper left), LYS (upper right), CS (lower left), and HYS (lower right) (**A**). The horizontal axis represents the number of alternative splicing events under each category, and the vertical axis represents the categories of alternative splicing events (**B**). Different coordinates represent different principal components, and the percentage indicates the contribution value of the corresponding principal component to the sample difference (**C**). The horizontal axis in the figure represents different samples; the vertical axis represents the logarithm of the sample expression level FPKM of the probability density (**D**). The novel genes were functionally enriched by KEGG and KOG (**E**).

Through base sequencing error rate analysis, it was found that the error rate of all samples was below 0.005% (Supplementary Material S2) and the samples remained stable during the sequencing process (Supplementary Material S3). Through sequencing quality control, a total of 107.89 Gb of Clean Data was obtained, and the percentage of Q30 bases in each sample was more than 95.46% (Supplementary Material S4). The alignment efficiency of the reads of each sample with the reference genome was between 95.61% and 97.28%, confirming the reliability of the data (Supplementary Material S5). The chromosomal coverage of the four stages throughout the genome shows that there is full coverage on all chromosomes (Figure 1A). The precursor mRNAs generated by gene transcription have multiple splicing methods. There were a total of 12 shearing methods for all the samples. Among them, the two types of cuts, Alternative 5′ first exon (transcription start site, TSS) and Alternative 3′ last exon (transcription terminal site, TTS), had the largest number. Next are Skipped exon (Skip) and Alternative exon ends (AE), and the number of other types of shearing occurred less frequently in all samples (Figure 1B). This project optimized the structures of 3657 genes (Supplementary Materials S6). A total of 6588 new genes were discovered by filtering out sequences with overly short encoded peptide chains (less than 50 amino acid residues) or those containing only a single exon (Supplementary Materials S7). The novel genes were sequentially aligned with the databases and were annotated. The quantities of new genes obtained by comparing various databases are as follows: COG (11), GO (584), KEGG (344), KOG (116), Pfam (227), Swiss-Prot (262), TrEMBL (1136), eggNOG (355), and nr (917). After removing duplicates, a total of 1243 novel genes were found and the Nr database was used, finding that most of the novel genes were distributed in Sus scrofa (Supplementary Materials S8). Principal component analysis (PCA) of the samples revealed that the repeatability among the samples was very high, while Pearson analysis found that the correlation between CS and HYS as well as LYS was very low, while the correlation between the SMS and LYS groups was relatively high (Figure 1C). By comparing the FPKM value density of each sample and the degree of dispersion, we found that the distribution of gene expression is similar (Figure 1D). GO enrichment analysis showed that novel genes were mainly enriched in physiological processes, including reproductive processes, developmental processes, etc. (Figure 1E).

**Figure 2:**
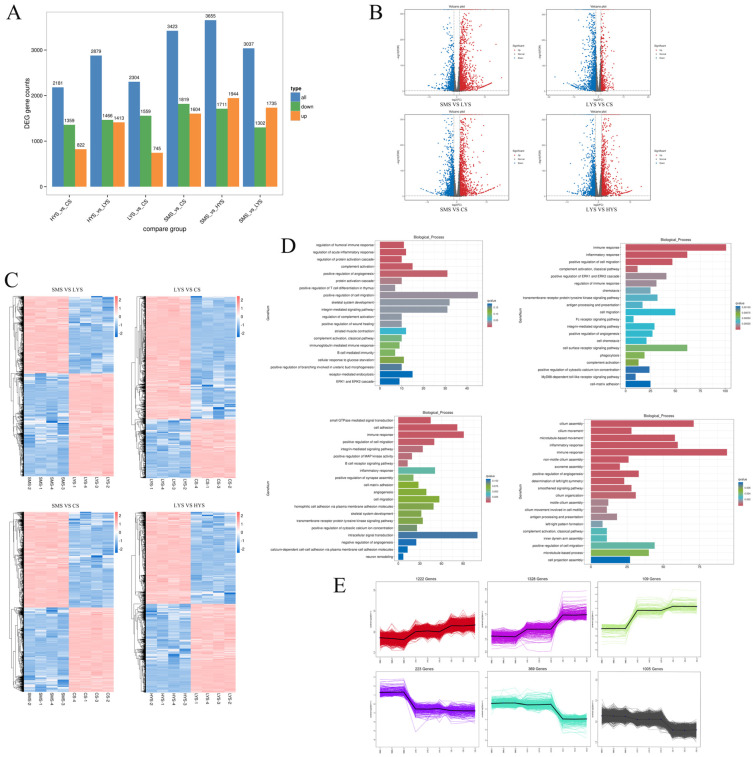
The horizontal axis represents different sets of DEGs: all DEGs (blue), upregulated genes (orange), and downregulated genes (green). The vertical axis represents the number of DEGs (**A**). Volcano plot demonstrating significant up- and downregulation of genes within four distinct groups (**B**). The expression level of the gene in the sample (**C**). The enrichment pathways of DEGs in each group in KEGG are displayed. SMS vs. LYS (upper left), LYS vs. CS (upper right), SMS vs. CS (lower left), and LYS vs. HYS (lower right) (**D**). Gene expression trend analysis was conducted on each sample from the three developmental stages (**E**).

A comparative analysis of uterine transcriptome results across four stages revealed the following DEG profiles. The SMS vs. LYS comparison identified 3037 DEGs, with 1735 upregulated and 1302 downregulated genes. For the LYS vs. CS comparison, 2304 DEGs were detected, comprising 745 upregulated and 1559 downregulated genes. The SMS vs. CS comparison showed 3423 DEGs, of which 1604 were upregulated and 1819 were downregulated. In SMS vs. HYS, a total of 3655 DEGs were observed, including 1944 upregulated and 1711 downregulated genes. Lastly, in LYS vs. HYS, there were 2879 DEGs in total, with 1413 upregulated and 1466 downregulated genes. It was found that 406 DEGs were differentially co-expressed at three different developmental stages (SMS, LYS, and CS) (Figure 2A,B). Gene Ontology (GO) enrichment analysis of two adjacent developmental sites as well as high-yield and low-yield DEGs revealed that the SMS vs. LYS group was primarily enriched in biological processes and molecular functions related to C-C chemokine binding and C-C chemokine receptor activity. Pathways associated with chemokine activity and cytokine receptor activity were also significantly enriched. In the LYS vs. CS group, DEGs were predominantly enriched in functions such as cytokine receptor activity, cytokine binding, chemokine activity, C-C chemokine receptor activity, and C-X-C chemokine receptor activity, among other related pathways. The SMS vs. CS group showed enrichment in pathways including calcium ion binding, C-C chemokine binding, microtubule binding, and chemokine binding. The HYS vs. LYS group was mainly enriched in ATP binding, ATP-dependent microtubule motor activity (minus end-directed), integrin binding, insulin-like growth factor (IGF) binding, and other associated pathways (Figure 2C). Through GSEA enrichment analysis, it was found that the results were mostly the same as those of GO enrichment analysis (Figure 2D and Supplementary Materials S9). Gene expression trend analysis was conducted on each sample from the three developmental stages, and it was found that the gene expression trend could be divided into 12 clusters in total. Among them, clusters 1, 7, and 11 showed a trend of increasing expression levels with individual age, while clusters 8, 10, and 12 showed a trend of gradually decreasing expression levels with age (Figure 2E).

### 3.2. Analysis of Differentially Accumulated Proteins (DAPs)

**Figure 3:**
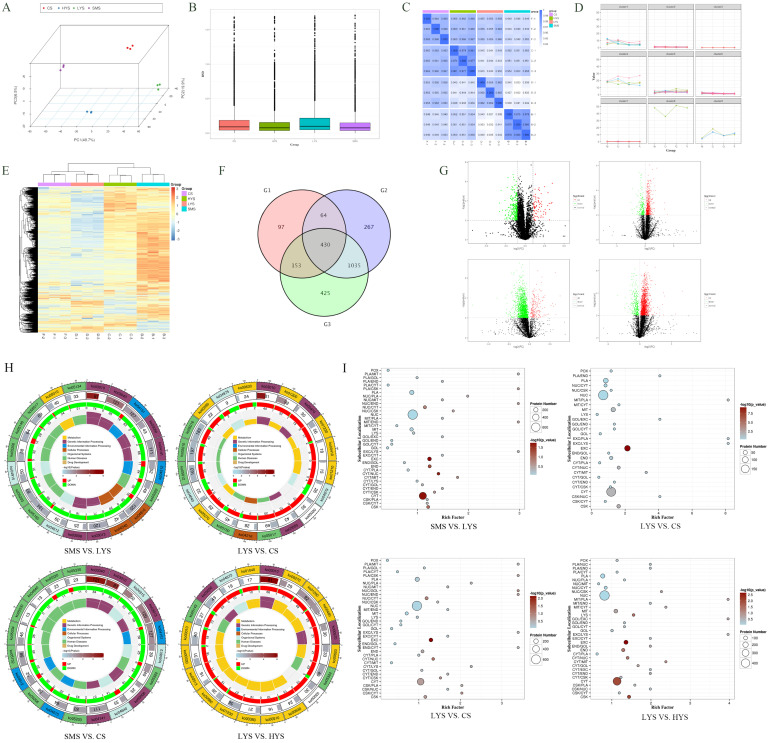
The percentage of the coordinate axes indicates the contribution proportion of this principal component to the sample difference (**A**). Relative Standard Deviation (RSD) refers to the ratio of the standard deviation to the mean (**B**). Sample correlation assessment is used to evaluate the similarity or difference between different samples, thereby identifying possible sample clustering, grouping, and other structures (**C**). The K-means clustering algorithm calculates the distance between each object and each sub-clustering center (**D**). The heat map of protein expression levels shows the changes in protein expression levels in different samples (**E**). Venn diagrams of differentially co-expressed proteins (G1-SMS vs. LYS, G2-LYS vs. CS, G3-SMS vs. CS) (**F**). Volcano plot demonstrating significant up- and downregulation of proteins within four distinct groups (**G**). KEGG enrichment analysis of the differential proteins in the four groups (**H**). The horizontal axis represents the enrichment factor, and the vertical axis represents subcellular localization. The larger the bubble, the more differential proteins are enriched, and the darker the bubble color, the more significant the enrichment (**I**).

PCA revealed significant inter-group differences, while the intra-group variations were relatively small (Figure 3A). The relative RSD distribution across all groups indicated a low level of data dispersion within each group, suggesting high biological reproducibility (Figure 3B). Furthermore, correlation analysis among all samples demonstrated that the correlation coefficients were consistently above 0.92, with most exceeding 0.96, indicating a very high degree of overall sample consistency (Figure 3C). K-means clustering of all proteins identified nine distinct clusters (Figure 3D). Heat map analysis was employed to visualize the expression levels of these proteins, revealing that the majority of proteins exhibited relatively high expression in the SMS group (Figure 3E). There were a total of 803 DAPs in the SMS vs. LYS comparison, of which 70 were upregulated and 733 were downregulated. In the LYS vs. CS comparison, a total of 35 DAPs were identified, with 21 upregulated and 14 downregulated. For the SMS vs. CS comparison, 499 DAPs were observed, including 441 upregulated and 58 downregulated proteins. In the LYS vs. HYS comparison, 299 DAPs were detected, comprising 51 upregulated and 258 downregulated proteins. Venn diagrams show that there were a total of 430 differentially co-expressed proteins at different developmental stages (SMS, LYS, and CS) (Figure 3F,G and Supplementary Material S10). Through KEGG enrichment analysis of the differential proteins in the four groups, it was found that the SMS vs. LYS differential proteins were mainly enriched in signaling pathways such as Ribosome and ECM–receptor interaction. The differential proteins of LYS vs. CS are mainly enriched in pathways such as Ribosome, Carbon metabolism, and Biosynthesis of amino acids, and at the same time, the differential proteins on these pathways are generally downregulated. The LYS vs. HYS differential protein KEGG pathway is mainly enriched in related pathways such as Ribosome, Glutathione metabolism, Cysteine, and methionine metabolism (Figure 3H).

The differential proteins in the SMS vs. LYS group showed the strongest enrichment trend in EXC (extracellular), and multiparous sows (LYS) demonstrated significant remodeling of the extracellular microenvironment. The enrichment trend of CYT/NUC (cytoplasm/nucleus) was obvious, reflecting the changes in the core processes of gene expression regulation and signal transduction. The obvious enrichment trends of CYT (cytoplasm) and END (endosome) also support the adaptive changes in basal metabolic processes (energy production, protein synthesis, etc.) in LYS; the differential proteins in the LYS vs. CS group were extremely significantly enriched in EXC (extracellular/extracellular), and significantly enriched in CYT/MIT (cytoplasm/mitochondria), MIT (mitochondria). LYS vs. HYS mainly showed a large number of proteins related to the cell nucleus (NUC) and cell membrane (PLA), and their quantity reached an extremely high statistical significance. Meanwhile, extracellular (EXC) secreted proteins, cytoskeletal (CSK), and cytoplasmic (CYT) proteins are also enriched to a certain extent (Figure 3I and Supplementary Materials S11).

### 3.3. Analysis of Differential Metabolites

**Figure 4:**
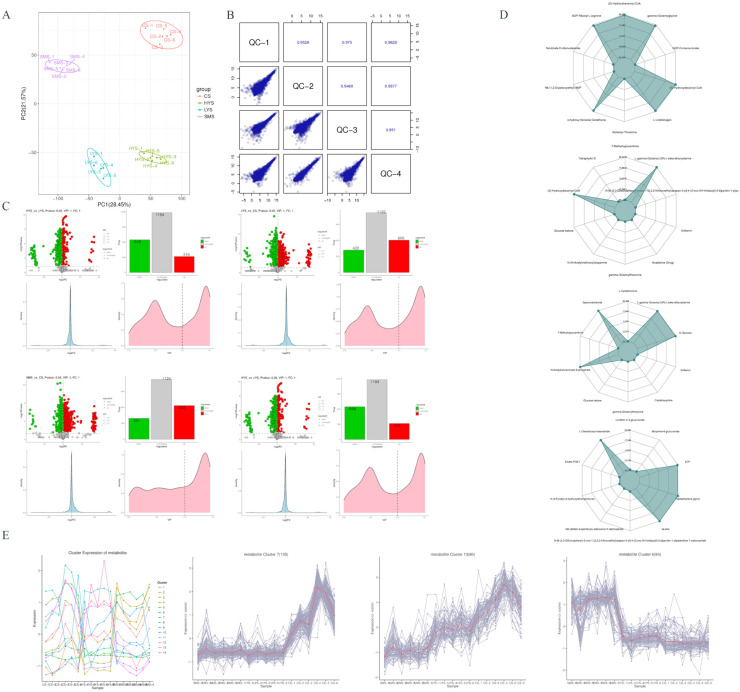
Principal component analysis of the sample (**A**). The diagonal squares represent the names of QC samples, the squares at the lower left of the diagonal are scatter plots of the correlation for QC samples, and the squares at the upper right of the diagonal represent the Spearman rank correlation coefficient r for QC samples. (**B**). Volcano plots of DEM (top left, log_2_FC vs. log10Pvalue), classification bar plots (top right, the number of down/unchanged/up), log_2_FC density plots (bottom left), VIP density plots (bottom right) among each group (**C**). The top 10 metabolites with the largest absolute log_2_FC values among each group are selected for radar chart display (**D**). K-means cluster and Sub Class represent the category numbers of metabolites with the same changing trend (**E**).

PCA of the samples at each stage revealed that based on all the measured variables, there were fundamental and statistically measurable differences in the overall pattern. The first and second principal components jointly explain approximately 50% of the overall variation (Figure 4A). Meanwhile, through the correlation scatter plot matrix of quality control (QC), it can be seen that the correlation coefficients between each pair of samples are very high (the lowest is 0.9468 and the highest is 0.9877), indicating that the reproducibility of the data is extremely good, and the distribution range of the points in all scatter plots is approximately the same. It is indicated that the signal strength distribution of all QC samples is similar, and no obvious signal drift occurs (Figure 4B). The metabolites were converted into Z-scores for quantitative analysis, and the top 30 differentially expressed metabolites in each group were subjected to statistical evaluation. Adenosine 3′-monophosphate was identified as a distinguishing metabolite in the SMS vs. LYS group. Significant differences were observed between N-Acetyl-S-(N-methylcarbamoyl) cysteine and 5-(3′-carboxy-3′-oxopropenyl)-4,6-dihydroxypicolinate, among others. In the LYS vs. CS group, notable differences were found between 4-[4-(3-hydroxyphenyl)-3-(4-methylphenyl)-6-oxo-1,4-dihydropyrrolo [3,4-d] pyrazol-5-yl] benzoic acid and Uridine monophosphate (UMP), as well as UDP-N-acetyl-alpha-D-glucosamine. In the SMS vs. CS group, significant differences were observed in Bicyclo-PGE2, Sabine, and Docosahexaenoic Acid ethyl ester. Additionally, in the LYS vs. HYS group, 1,2-Naphthoquinone and 2′-O-trans-p-coumaroylastragalin exhibited significant variation (Supplementary Materials S12). The visualization of differential metabolites indicates that there are 1044 differentially expressed metabolites in the SMS vs. LYS comparison, with 571 upregulated and 473 downregulated. The top 5 differential metabolites include: N-Acetyl-S-(N-methylcarbamoyl) cysteine, 2′-O-trans-p-coumaroylastragalin, 1,2-Naphthoquinone, Glutarate 4-[4-(3-hydroxyphenyl)-3-(4-methylphenyl)-6-oxo-1,4-dihydropyrrolo [3,4-d] pyrazol-5-yl] benzoic acid. In the LYS vs. CS comparison, a total of 1387 differential metabolites were identified, of which 566 were upregulated and 821 were downregulated. The top five differential metabolites are UDP-N-acetyl-alpha-D-glucosamine, N-Acetyl-S-(N-methylcarbamoyl) cysteine, Furegrelate, 4-(beta-D-Glucosyloxy) benzoate, and ADP. For the SMS vs. CS group, there are 1397 differential metabolites, including 574 upregulated and 823 downregulated. The top five differential metabolites are Oxprenolol, Stearidonoyl glycine, 6-Thioxanthine 5′-monophosphate, Leukotriene B5, and 2-[[4-[(2-amino-4-oxo-3H-pteridin-6-yl) methylamino] benzoyl] amino]-5-methoxy-5-oxopentanoic acid. In the LYS vs. HYS comparison, 1304 differential metabolites were detected, with 433 upregulated and 871 downregulated. The top five differential metabolites were 2″-O-trans-p-Coumaroylastragalin, Trp-Tyr, N-Arachidonoyl Proline, Pseudooxynicotine, and Etimicin (Figure 4C). By calculating the corresponding ratios of the quantitative values of differential metabolites in each group, the top 10 metabolites in each group were identified. In the SMS vs. LYS group, (S)-Hydroxyhexanoyl-CoA, gamma-glutamyl-glutamic acid, 4-hydroxy-2-nonenal glutathione, and ADP-ribosyl-L-arginine were significantly upregulated. In the LYS vs. CS group, (S)-hydroxyhexanoyl-CoA and L-gamma-glutamyl-(3R)-L-beta-ethynylserine were the most differentially expressed metabolites. In the SMS vs. CS group, significant differences were observed in N-acetylneuraminate 9-phosphate, L-gamma-glutamyl-(3R)-L-beta-ethynylserine, specnuezhenide, and D-glucose. Among the LYS vs. HYS group, XTP, heptaethylene glycol, L-olivosyl-oleandolide, and Ile-Phe were among the metabolites with the greatest differences (Figure 4D). A trend analysis of metabolite expression levels was conducted on all samples from three different developmental stages, and it was found that they could be divided into a total of 14 clusters. Among them, the metabolites in cluster 7 and cluster 13 showed an increasing trend of expression levels over time, while in cluster 6, the expression levels gradually decreased with age (Figure 4E).

### 3.4. Multi-Omics Integrated Analysis

**Figure 5:**
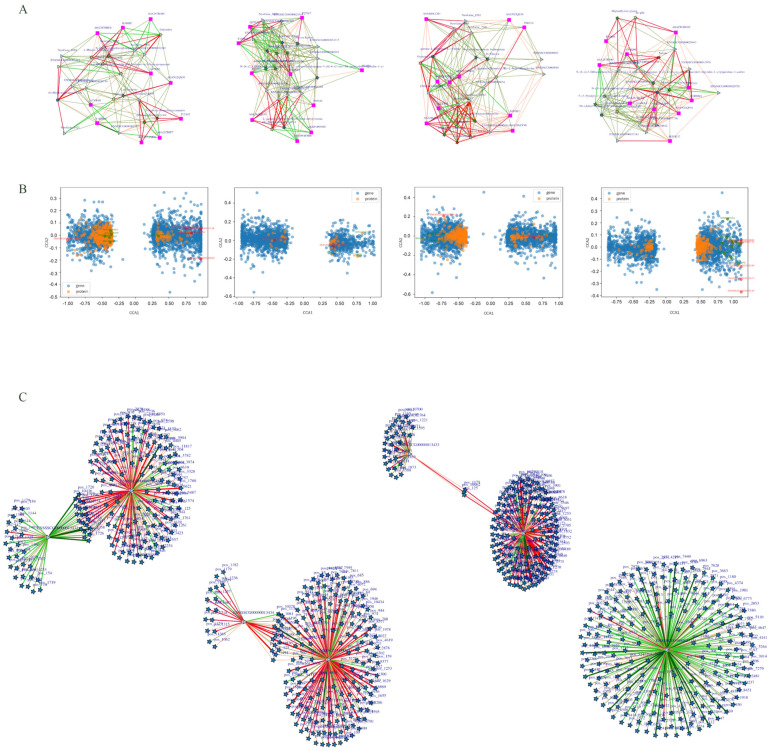
Regulatory network analysis was performed for four pairwise comparison groups (SMS vs. LYS, LYS vs. CS, SMS vs. CS, LYS vs. HYS) (**A**). Restricted correspondence analysis to analyze the associations of DEG and DAP among the groups (**B**). Spearman correlation analysis was performed to evaluate the strength of associations between DEGs and DEMs in each pairwise comparison group (**C**).

To explore the potential associations among the three omics datasets in relation to DEGs, regulatory network analysis was conducted based on DAP and DEMs in the SMS vs. LYS group. The analysis identified several key molecular entities, including the DEGs ENSSSCG00000003732 (MEP1B), ENSSSCG00000003417 (DISP3), ENSSSCG00000032503, ENSSSCG00000020750, and ENSSSCG00000022251 (UABP-2); the DAP A7VK00 (Interferon-induced GTP-binding protein Mx2), F1RHM1 (Methionine synthase), A0A287AM28 (NDRG family member 4), A0A287BJF7 (Sphingomyelin phosphodiesterase 4), O19062 (C-reactive protein), and A0A287B300 (Vacuolar protein sorting-associated protein 53 homolog); and the differentially expressed metabolites N-(N-acetylmethionyl) dopamine, 3-[(4R)-4-hydroxycyclohexa-1,5-dien-1-yl]-2-oxopropanoate, GDP-D-mannuronate, and Endoxifen. These components collectively form a regulatory network. In the LYS vs. CS group, the following genes were identified: ENSSSCG00000006590 (S100A8), ENSSSCG00000024314 (FGG), ENSSSCG00000036233, and ENSSSCG00000026260 (ATP13A5). Additionally, DAPs included F1SRK6 (Endoplasmin), P02101 (Hemoglobin subunit epsilon), P27917 (Apolipoprotein C-III), Q29529 (Carbonyl reductase [NADPH] 2), and P27594 (Interferon-induced GTP-binding protein Mx1). The differentially regulated metabolites, such as Volkenin, gamma-Glutamylthreonine, and Glucose ketone, among others, collectively constitute the regulatory network. DEGs identified in the SMS vs. CS group included ENSSSCG00000032503 (CBD139), ENSSSCG00000012835 (MUC6), ENSSSCG00000017076 (NMUR2), and ENSSSCG00000003417 (DISP3). Differentially abundant proteins such as A0A287AM28 (NDRG family member 4), A0A480LCB3 (Formin like 1), A0A5G2QS30 (Adhesion G protein-coupled receptor A2), and Q2TJA5 (Aldo-keto reductase) were also observed. Additionally, differential metabolites including N-acetylneuraminate 9-phosphate, D-glucose, gamma-glutamylthreonine, volkenin, tryptophyl-threonine, and glucose ketone were detected. Together, these molecular differences contribute to the construction of an integrated regulatory network. Differential genes were found in the regulatory network of the LYS vs. HYS group: ENSSSCG00000029043(NXPE2), ENSSSCG00000013976, ENSSSCG00000035698, ENSSSCG00000006590(S100A8), ENSSSCG00000037736, etc. The differential proteins include A0A8W4FG52 (LAS1 like ribosome biosis factor), P16469 (Polyunsaturated fatty acid lipoxygenase ALOX15), A0A287B300 (Vacuolar protein sorting-associated protein 53 homolog), A0A287BCT2 (Acyl-coenzyme A thioesterase 4), A0A286ZZP8 (Signal recognition particle subunit SRP72), etc. The differential metabolites include Nonate, XTP, Morphine 6-glucuronide, N-(N-acetylmethionyl) dopamine, ile-phe, etc. (Figure 5A).

To investigate the relationship between DEG and DAP, a restricted correspondence analysis was performed across four groups. The results indicated that among the top 50 genes identified in the SMS VS. LYS group, several were associated with uterine growth and development, including ENSSSCG00000000692 (PIANP), ENSSSCG00000027901 (CLDN22), ENSSSCG00000001849 (ANPEP), and ENSSSCG00000009738 (GALNT9). Corresponding DAPs included A0A287A3N0, A0A287A1M3, A0A287A2R7, A0A287A9Y6, A0A287A4W5, A0A287A0K0, and A0A287A2M4. In the LYS VS. CS group, genes related to uterine growth and development among the top 50 genes were ENSSSCG00000004755 (DLL4). The corresponding proteins were A0A5G2QE35, F1SG41, F1SHC0, and Q29529. In the SMS vs. CS group, the genes ENSSSCG00000008119 and ENSSSCG00000022331 were found to be associated with uterine growth and development, with corresponding proteins A0A480LCB3, B3F0B7, A0A287AM28, A0A286ZY90, and A0A5G2QS30. In the LYS vs. HYS group, the genes ENSSSCG00000029744, ENSSSCG00000029043, ENSSSCG00000022175, ENSSSCG00000009735, and ENSSSCG00000002830 were identified as being related to uterine development. The top 10 associated proteins were P16469, A0A286ZZP8, F1RQ08, F1RHM1, A0A5G2QF41, M3VK37, A0A8W4FG52, A0A287B300, A0A287BCT2, and I3LHZ9 (Figure 5B).

Spearman correlation analysis revealed that in the SMS VS. LYS group, the ENSSSCG00000013434 (PLK5) and ENSSSCG00000013433 (ADAMTSL5) genes were strongly correlated with multiple metabolites. In the LYS vs. CS group, the PLK5 and ENSSSCG00000023351 (PLA2G4A) genes were strongly correlated with a variety of metabolites. In the LYS vs. HYS group, PLA2G4A was in the central position and negatively correlated with multiple metabolites (Figure 5C).

### 3.5. RT-qPCR Quantification of mRNAs

**Figure 6:**
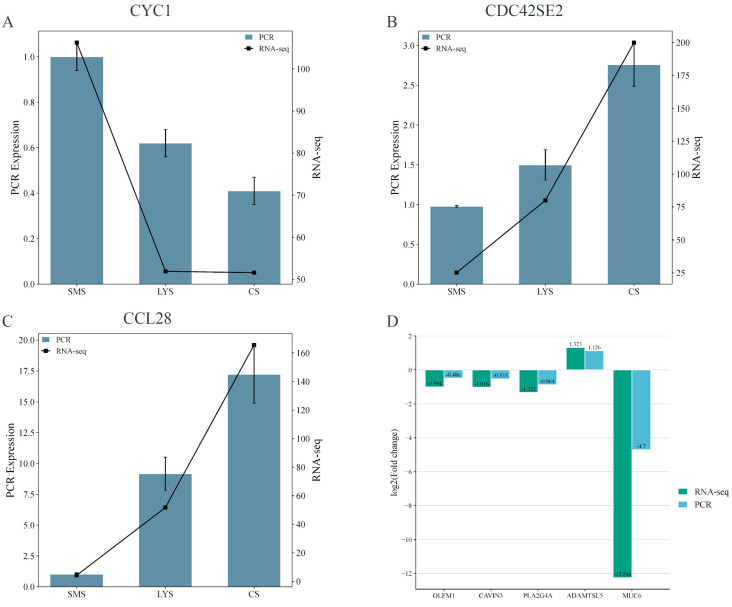
Transcription patterns of CYC1 (**A**), CDC42SE2 (**B**), CCL28 (**C**) and OLFM1, CAVIN3, PLA2G4A, ADAMTSL, MUC6 (D) compared to expression patterns in the RNA-seq.

Eight genes were subjected to validation experiments, including co-expressed genes across three developmental stages (CYC1, CDC42SE2, and CCL28); DEGs between LYS and HYS (OLFM1, CAVIN3, and PLA2G4A); and DEGs between SMS and CS (ADAMTSL5 and MUC6). The results showed that the experimental data exhibited consistent expression trends with the sequencing data, confirming the reliability of the sequencing results (Figure 6).

## 4. Discussion

### 4.1. Transcriptomic Profiles Reveal Core Pathways of Dynamic Uterine Function Regulation

Transcriptomic analysis systematically delineated the dynamic changes in uterine gene expression across four reproductive states of LW pigs, with rigorous quality control confirming the reliability of the data (Figure 1A), while Q30 base percentages exceeded 95.46%—collectively meeting the standards for deep functional analysis. Notably, 12 AS types were detected, with TSS and TTS as dominant ones (Figure 1B). In the CS stage, splicing disorders may worsen inflammation, matching its “extracellular inflammatory characteristics” (Figure 3I). DHA ethyl ester needs carboxylesterase hydrolysis to release anti-inflammatory free DHA: stored as ethyl ester in SMS (avoids premature immunosuppression), enzyme activity rises in HYS, and its content drops in CS (insufficient anti-inflammation causes decline) (Figure 4C). XTP, synthesized by IMPDH/GK enzymes, is highly expressed in HYS. It activates DNA synthase more efficiently than ATP to support multi-embryo placental angiogenesis, suggesting it is a high-fecundity metabolic marker (Figure 4D) [12].

Beyond quality validation, structural refinement of 3657 genes and identification of 6588 novel genes (1243 functionally annotated) further expanded the porcine uterine transcriptomic database. Functional enrichment of these novel genes provided critical clues: COG analysis highlighted enrichment in energy production and conversion (supporting uterine cell proliferation and nutrient supply) [13], and signal transduction mechanisms (regulating embryo–uterine crosstalk) [14]; GO analysis further linked them to reproductive processes and developmental processes (Figure 1E). These findings imply that novel genes may play underappreciated roles in uterine maturation and pregnancy adaptation.

Among inter-group DEGs, the SMS vs. CS comparison exhibited the most dramatic changes (5718 DEGs, including 2967 upregulated and 2751 downregulated genes), followed by SMS vs. LYS (5520 DEGs) and LYS vs. CS (5073 DEGs). This pattern indicates that the transition from sexual maturity to culled involves the most extensive transcriptional reprogramming—consistent with the uterus undergoing structural and functional remodeling aging processes [15]. For HYS vs. LYS (5073 DEGs), enrichment in ATP-dependent microtubule motor activity (facilitating cytoskeletal remodeling for embryo implantation) [16] and IGF binding (enhancing placental nutrient transport) remained critical [17], but additional context from hub genes (identified via Spearman correlation) adds depth: genes such as PLK5 and ADAMTSL5 (SMS vs. LYS) and PLA2G4A (LYS vs. HYS, LYS vs. CS) were strongly correlated with multiple metabolites, suggesting they act as “transcriptional–metabolic bridges” to coordinate uterine function. PLA2G4A (a phospholipase gene) was negatively correlated with metabolites in HYS vs. LYS, potentially suppressing excessive inflammatory responses to maintain a favorable implantation microenvironment [18].

In contrast, DEGs associated with CS underscored a “decline signature”: LYS vs. CS DEGs were enriched in cytokine receptor activity and C-C chemokine binding (consistent with chronic immune cell infiltration), while SMS vs. CS DEGs showed downregulation of calcium ion binding (disrupting uterine smooth muscle contraction and cell proliferation). Notably, the 406 co-expressed DEGs across SMS, LYS, and CS (Figure 2B) may represent “core regulatory genes” governing uterine lifespan—with their dynamic expression providing a genetic timeline of uterine function from maturation to decline.

### 4.2. Proteomic Differences Reflect the Structural and Functional Fit of the Uterine Functional State

Proteomic analysis validated transcriptional trends at the functional protein level, with high data reliability supported by low intra-group RSD, sample correlation coefficients > 0.92 (most > 0.96), and clear inter-group separation via PCA (Figure 3A–C). A key observation was that the majority of proteins exhibited higher expression in the SMS group (Figure 3E), indicating that sexually mature uteri maintain robust protein synthesis activity—likely to support pre-pregnancy preparation (e.g., ECM remodeling and nutrient storage) before the first parity.

Functional enrichment of DAPs revealed stage-specific adaptation: SMS vs. LYS (803 DAPs, 70 up/733 down): DAPs were enriched in Ribosome (enhanced protein synthesis) and ECM–receptor interaction (uterine stromal remodeling). This aligns with the transition from “pre-pregnancy readiness” to “post-parity maintenance”—the downregulation of 733 DAPs may reflect a shift from extensive tissue growth to targeted functional adaptation (e.g., reducing unnecessary protein synthesis to conserve energy for subsequent pregnancies [19]); LYS vs. CS (35 DAPs, 21 up/14 down). Despite the small number, DAPs were significantly enriched in Ribosome, Carbon metabolism, and Biosynthesis of amino acids—with universal downregulation indicating impaired basal metabolic capacity in CS uteri [20]. Subcellular localization analysis further revealed extreme enrichment of DAPs in the extracellular (EXC) compartment (*p* < 0.001), alongside significant enrichment in cytoplasm/mitochondria (CYT/MIT) and mitochondria (MIT) (Figure 3I) [21]. This suggests that chronic inflammation (driven by EXC DAPs such as F1SRK6/Endoplasmin, a chaperone linked to stress responses) and mitochondrial dysfunction collectively drive uterine functional decline [22]; LYS vs. HYS (299 DAPs, 51 up/258 down): DAPs were dominated by nuclear (NUC) and cell membrane (PLA) proteins, including A0A8W4FG52 (LAS1-like ribosome biogenesis factor, supporting ribosome assembly for protein synthesis) and P16469 (ALOX15, a polyunsaturated fatty acid lipoxygenase that regulates anti-inflammatory lipid mediators) [23]. The high proportion of NUC proteins suggests enhanced transcriptional regulation in HYS uteri—for example, via modulating genes involved in angiogenesis or nutrient transport—while PLA proteins may strengthen cell adhesion between trophoblasts and the endometrium, supporting multi-embryo implantation [24].

Notably, 430 co-expressed DAPs across SMS, LYS, and CS (Figure 3F) highlight conserved protein-level regulators of uterine lifespan. For instance, consistent downregulation of ECM-related DAPs from SMS to CS may reflect progressive loss of uterine structural integrity—an observation that cross-validates transcriptomic findings of ECM-receptor pathway enrichment in early stages.

### 4.3. Metabolomic Characteristics Reveal the Material Basis for Maintaining Uterine Function

As the “phenotypic executor,” differential expressed metabolites (DEMs) directly reflect the uterine microenvironment’s nutritional status and redox balance. Rigorous quality control—including QC sample correlation coefficients > 0.9468 and no significant signal drift (Figure 4B)—ensured metabolomic data reliability. The large number of DEMs (e.g., 1397 in SMS vs. CS, 1387 in LYS vs. CS) underscored extensive metabolic reprogramming across reproductive stages.

Stage-specific metabolic signatures were evident: SMS vs. LYS: Key upregulated DEMs included (S)-Hydroxyhexanoyl-CoA (a fatty acid β-oxidation intermediate, supporting energy production for uterine remodeling) and gamma-glutamyl-glutamic acid (a glutathione precursor, enhancing redox homeostasis). These metabolites align with transcriptomic/proteomic trends of increased protein synthesis and ECM remodeling—providing energy and antioxidant protection during the transition to first parity. Adenosine 3′-monophosphate (a nucleotide metabolite) was also a distinguishing DEM, potentially regulating uterine cell proliferation via purine metabolism [25]; LYS vs. HYS: the most impactful DEMs were XTP (a purine energy donor, upregulated in HYS) and Ile-Phe (a dipeptide, downregulated in HYS). XTP’s high expression in HYS directly supports the transcriptomic enrichment of ATP-dependent pathways, providing additional energy for placental nutrient transport and multi-embryo development [26]. Ile-Phe downregulation may reflect increased utilization of essential amino acids by developing embryos—consistent with proteomic observations of enhanced nutrient-related protein expression in HYS [27]; LYS vs. CS: (S)-Hydroxyhexanoyl-CoA (downregulated) and L-gamma-glutamyl-(3R)-L-beta-ethynylserine (upregulated) were top DEMs. The decline in fatty acid metabolism intermediates further confirms impaired energy production in CS uteri, while upregulation of gamma-glutamyl metabolites may indicate a compensatory attempt to maintain redox balance (ultimately failing, as seen in transcriptomic inflammation signatures) [28]. UDP-N-acetyl-alpha-D-glucosamine (a glycosylation precursor, downregulated) also suggests reduced ECM glycosylation—weakening uterine structural integrity [29]; SMS vs. CS: Docosahexaenoic Acid (DHA) ethyl ester (downregulated in CS) remained critical, as DHA inhibits excessive inflammation and promotes placental angiogenesis. Its decline in CS aligns with chronic endometritis phenotypes. Additionally, Leukotriene B5 (an inflammatory mediator, upregulated in CS) and Stearidonoyl glycine (a lipid metabolite, downregulated in CS) further highlight the “inflammatory-nutritional imbalance” driving uterine decline [30].

Metabolite trend clustering (14 clusters) reinforced these patterns: clusters 7/13 (increasing with age) included inflammatory mediators (e.g., Leukotriene B5), while cluster 6 (decreasing with age) included nutrient metabolites (e.g., DHA ethyl ester). This three-way alignment with transcriptomic/proteomic trends confirms that “inflammatory intensification” and “nutrient depletion” are core drivers of uterine aging.

### 4.4. Multi-Omics Joint Analysis of the Molecular Network Regulating Uterine Function

Integrated analysis of transcriptomic, proteomic, and metabolomic data uncovered synergistic regulatory mechanisms that single-omics studies could not capture—revealing specific “gene–protein–metabolite” modules for each reproductive stage.

The SMS vs. LYS module centered on DEGs (ENSSSCG00000003732/MEP1B, ENSSSCG00000003417/DISP3), DAPs (A7VK00/Mx2, an interferon-induced GTPase; F1RHM1/Methionine synthase, regulating amino acid metabolism), and DEMs (N-(N-acetylmethionyl) dopamine, a signal transduction metabolite). MEP1B (a metalloprotease gene) may modulate ECM remodeling via DAPs like Mx2 (supporting immune homeostasis) [31], while DISP3 (a hedgehog signaling gene) could coordinate metabolite synthesis (e.g., dopamine derivatives) to optimize the pre-implantation microenvironment [32]; LYS vs. CS module key molecules included DEGs (ENSSSCG00000006590/S100A8, a pro-inflammatory gene; ENSSSCG00000024314/FGG, a coagulation factor), DAPs (F1SRK6/Endoplasmin, a stress chaperone; P27917/Apolipoprotein C-III, regulating lipid metabolism), and DEMs (Volkenin, gamma-Glutamylthreonine). S100A8 upregulation (linked to DAP F1SRK6) drives extracellular inflammation, while FGG downregulation may impair uterine vascular function—exacerbated by gamma-Glutamylthreonine decline (disrupting redox balance). This module explains the “inflammatory-metabolic collapse” in CS uteri [33]; LYS vs. HYS: The core module featured DEGs (ENSSSCG00000029043/NXPE2, a signaling gene; ENSSSCG00000035698/VEGFA-like), DAPs (A0A8W4FG52/LAS1-like, supporting ribosome biogenesis; P16469/ALOX15, regulating anti-inflammatory lipids), and DEMs (XTP, Ile-Phe). NXPE2 may enhance endometrial receptivity via ALOX15-mediated lipid signaling, while VEGFA-like genes coordinate with XTP (energy) and Ile-Phe (amino acids) to support angiogenesis and embryo nutrient supply, collectively explaining HYS’s high litter size.

The DEG-DAP association discovered by restricted correspondence analysis (RCA) further focuses on key regulatory nodes. For SMS vs. LYS, DEGs like ENSSSCG00000000692/PIANP (ECM remodeling) and ENSSSCG00000027901/CLDN22 (tight junction formation) correlated with DAPs such as A0A287A3N0—highlighting the role of “ECM-junction” modules in pre-pregnancy adaptation. For LYS vs. HYS, DEGs like ENSSSCG00000029744 (angiogenesis) correlated with DAPs A0A8W4FG52/LAS1-like—linking ribosome biogenesis to vascular function. Hub genes PLA2G4A, PLK5, and ADAMTSL5 showed strong associations with multiple metabolites across comparisons. PLA2G4A, in particular, was a central node in LYS vs. HYS (negatively correlated with inflammatory metabolites) and LYS vs. CS (positively correlated with pro-inflammatory metabolites)—suggesting it acts as a “switch” regulating uterine inflammation. These genes are priority targets for functional validation, as their modulation could potentially reverse uterine decline or enhance high-yield performance.

In summary, multi-omics integration reveals that uterine function is governed by stage-specific “gene-protein-metabolite” modules: maturation (energy–redox balance), low yield (structural adaptation), high yield (angiogenesis–nutrient coordination), and decline (inflammatory-metabolic collapse). These insights provide a precise molecular map for optimizing sow reproductive lifespan in commercial production.

### 4.5. Research Limitations

Although this design can initially construct stage-specific molecular maps, the small biological duplicate sample size in multi-omics analysis (*n* = 3/group) may limit its ability to distinguish some weak differential signals. Subsequent studies need to further verify this by increasing the repetition volume.

The “molecular associations” identified through the multi-omics integrated analysis in this study are correlational conclusions, which can only reflect the co-occurrence relationship between molecular expression and the physiological state of the uterus, and cannot directly prove the causal regulatory role. Subsequent studies need to combine cell experiments (such as primary uterine stromal cell culture) and animal models (such as gene-edited pigs) to verify the causal regulatory relationships of core molecules through intervention experiments.

This study only collected uterine tissue for multi-omics analysis and did not simultaneously obtain tissue samples closely related to reproductive function, such as ovaries and placenta. As the “target organ” for reproductive regulation, the molecular state of the uterus is regulated upstream by ovarian hormones (such as estrogen and progesterone) and also works in coordination with the nutrient transport function of the placenta. Relying solely on uterine tissue cannot fully elucidate the multi-organ regulatory network of “ovary—uterus—placenta.” Future research needs to supplement multi-omics data from tissues such as ovaries and placenta to construct a multi-organ coordinated regulation model and enhance the systematic understanding of the regulatory mechanisms of reproductive function.

## 5. Conclusions

This study employed integrated transcriptomic, proteomic, and metabolomic approaches to explore uterine function dynamics across four reproductive stages (SMS, LYS, HYS, and CS) in LW pigs. Rigorous quality control ensured data reliability. Transcriptomics identified 12 alternative splicing types and 1243 annotated novel genes, with SMS vs. HYS showing the most DEGs enriched in implantation/nutrient pathways. Proteomics revealed stage-specific DAPs, such as high SMS protein synthesis and CS extracellular inflammatory DAPs. Metabolomics uncovered signature metabolites (e.g., XTP, SMS DHA ethyl ester) linking metabolism to reproduction. Multi-omics integration deciphered stage-specific “gene–protein–metabolite” modules and hub genes like PLA2G4A. These findings fill gaps in LW pig uterine research and provide a molecular map for optimizing sow reproductive lifespan in commercial production.

## Figures and Tables

**Table 1:** Primer sequences information.

Genes	Primer	Length (bp)
CYC1	F: GGCTGAGGAGGTGGAGGTTCAA	163
	R: TCGCACGATGTAGCTGAGGTCA	
CDC42SE2	F: TCGGTCAGCTCCATCCAGAACC	100
	R: ATCCTGCCTTCGTGTCCACAAG	
CCL28	F: GCTGCTGCACTGAGGTTTCACA	80
	R: ATCCGCTCTCTGAAGGCGACAT	
OLFM1	F: ACCTGAAGACCGAGAGCATCCT	150
	R: CGTTCTGGTTGGTGGCGTAGAC	
CAVIN3	F: CCACGACACGACGAGCAACA	186
	R: TCAGCCTCCTCCTTGAAGAGCA	
PLA2G4A	F: TGGTGGACAGTGGCCTCACATT	121
	R: AACGGAGGACTGGAGTCGCTTG	
ADAMTSL5	F: CCACTATTGCGGCAGCGACTT	118
	R: CAGAGGCGAGCGGTTCTTGTAG	
MUC6	F: ACTCCGCACTGGACCGAAGAA	130
	R: CCGTTCCGCTGGTGGTTGTT	
GAPDH	F: GGTGAAGGTCGGAGTGAACG	195
	R: CCTGGAAGATGGTGATGGGAT	

## Data Availability

The original contributions presented in this study are included in the article/Supplementary Material. Further inquiries can be directed to the corresponding authors.

## Supplementary Materials

The following supporting information can be downloaded at: https://www.mdpi.com/article/10.3390/biology14111589/s1, Supplementary Materials S1: Ration ratio; Supplementary Materials S2: Base sequencing error rate analysis; Supplementary Materials S3: the samples remained stable during the sequencing process; Supplementary Materials S4: sequencing quality control; Supplementary Materials S5: The alignment efficiency of the reads of each sample with the reference genome; Supplementary Materials S6: This project optimized the structures of genes; Supplementary Materials S7: New genes were discovered by filtering out sequences; Supplementary Materials S8: The novel genes were sequentially aligned with the databases and were annotated; Supplementary Materials S9: GSE enrichment; Supplementary Materials S10: Protein expression; Supplementary Materials S11: Four stages of differential protein pathway enrichment; Supplementary Materials S12: z-score analysis of differential metabolites in four stages.
