# Supplementary material for: Integrated Multi-Omics Analysis Reveals Stage-Specific Molecular Modules Regulating Uterine Function and Fecundity in Large White Pigs Across Reproductive Lifespan
Source: Biology (Basel). 2025 Nov 13;14(11):1589. doi: 10.3390/biology14111589 (PMC12649833; doi:10.3390/biology14111589)
Supplement: Supplementary file 1 [file biology-14-01589-s001.zip › Supplementary Materials S1 Ration ratio.pdf]

Raw material name	proportion (%)	Nutritional level	Measured value
Corn	62.0	Digestive energy (MJ/kg)	13.6
Soybean meal	18.0	Crude protein (%)	15.2
Bran	12.0	Calcium (%)	0.88
Soybean oil	2.5	Available phosphorus (%)	0.32
Stone powder	1.5		
Calcium hydrogen phosphate	1.2		
Premix	2.8		

supplied(per kg diet)： Vitamin A: 500,000 IU; Vitamin D3:100,000 IU Vitamin K3:200 mg Vitamin B1:50 mg Vitamin B2:150 mg Vitamin B6:80 mg Vitamin B12:1.5mg Biotin: 5 mg Choline: 150 g; Copper: 2000 mg; Iron: 15,000 mg; Zinc: 15,000 mg; Manganese: 8000 mg; Iodine: 200 mg; Ethoxyquinoline (antioxidant) : 500 mg
