# Supplementary figures and images for: Integrated Multi-Omics Analysis Reveals Stage-Specific Molecular Modules Regulating Uterine Function and Fecundity in Large White Pigs Across Reproductive Lifespan
Source: Biology (Basel). 2025 Nov 13;14(11):1589. doi: 10.3390/biology14111589 (PMC12649833; doi:10.3390/biology14111589)

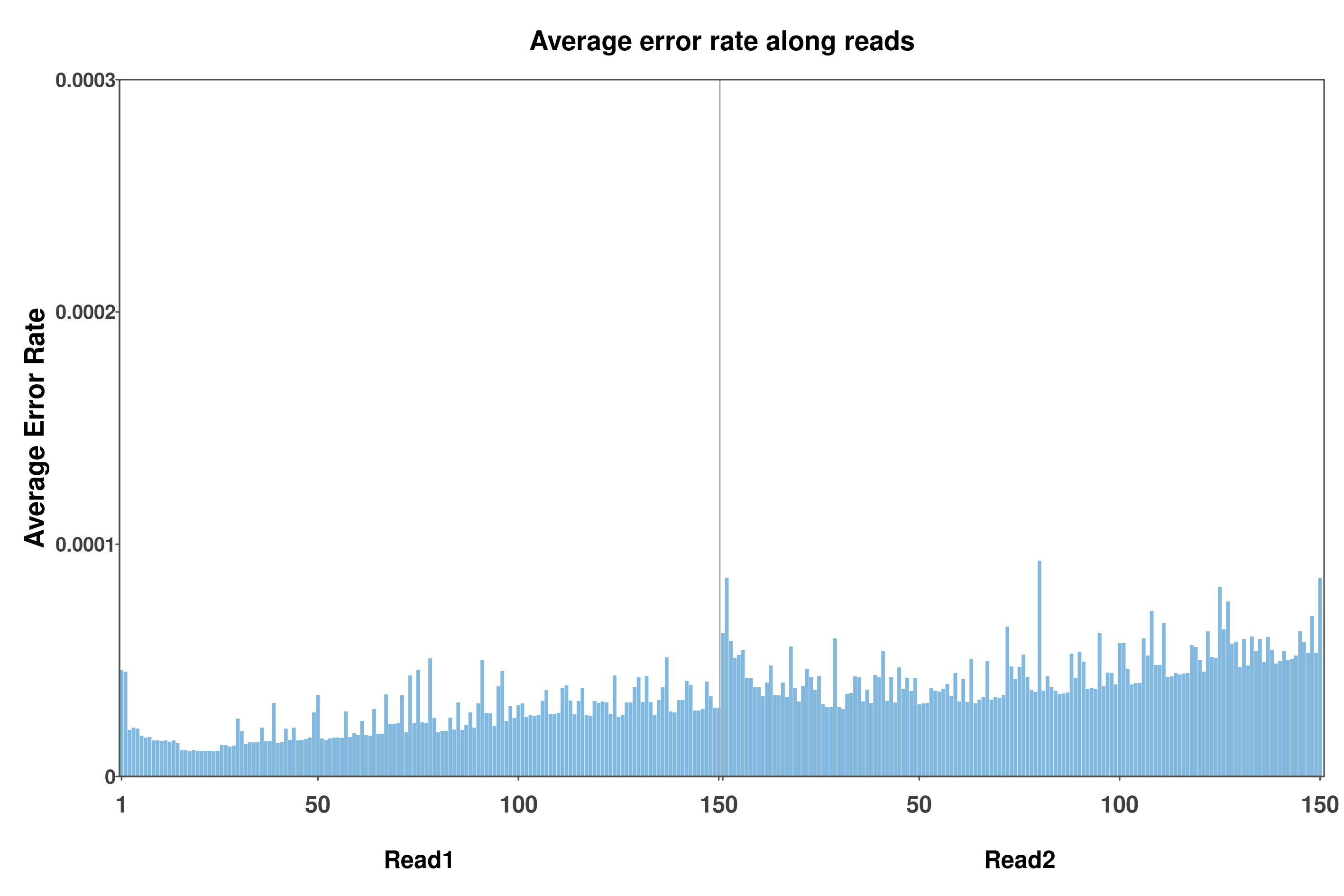

Supplement: Supplementary file 1 [file biology-14-01589-s001.zip › Supplementary Materials S2-Base sequencing error rate analysis/CS-1.quality.png]

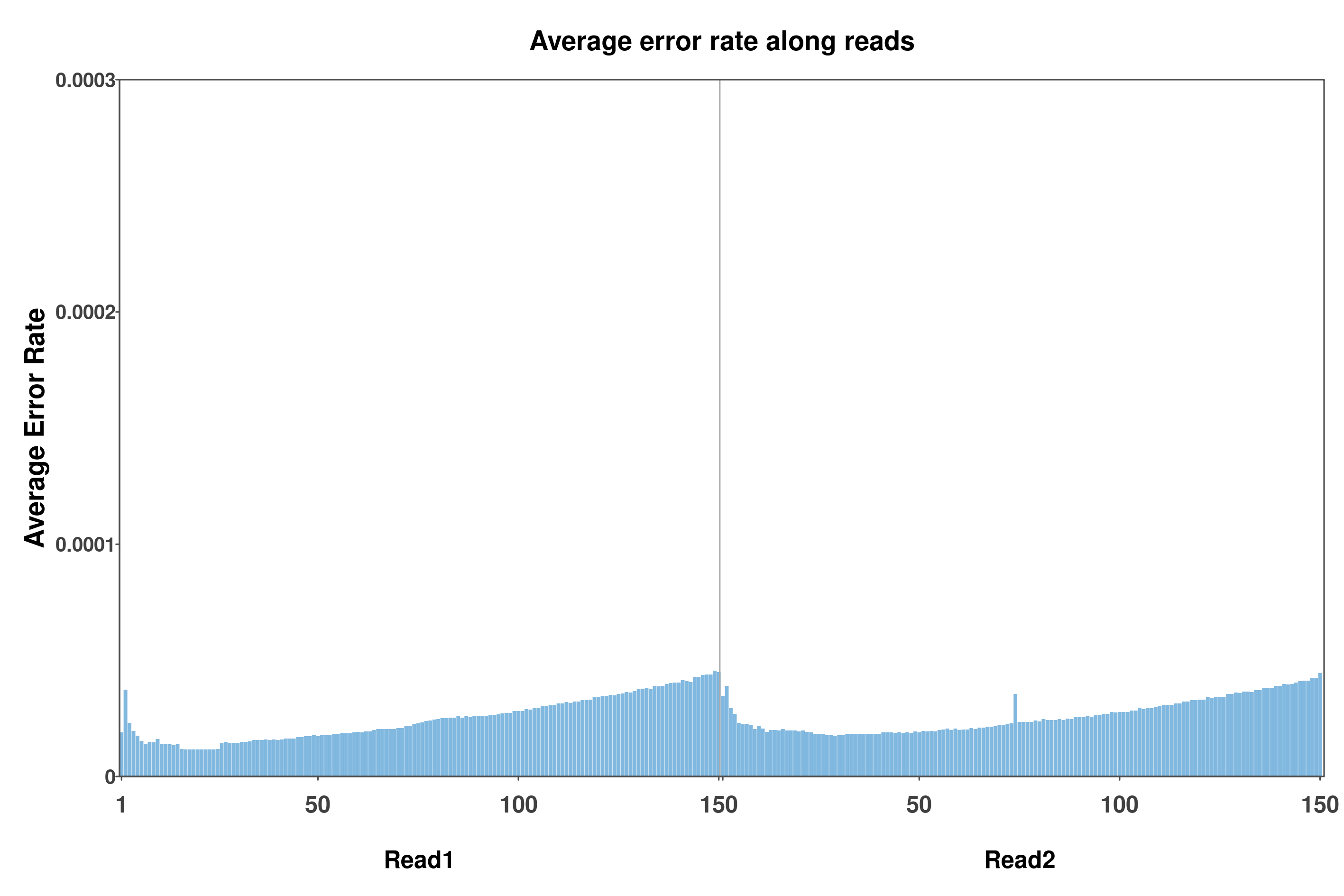

Supplement: Supplementary file 1 [file biology-14-01589-s001.zip › Supplementary Materials S2-Base sequencing error rate analysis/CS-2.quality.png]

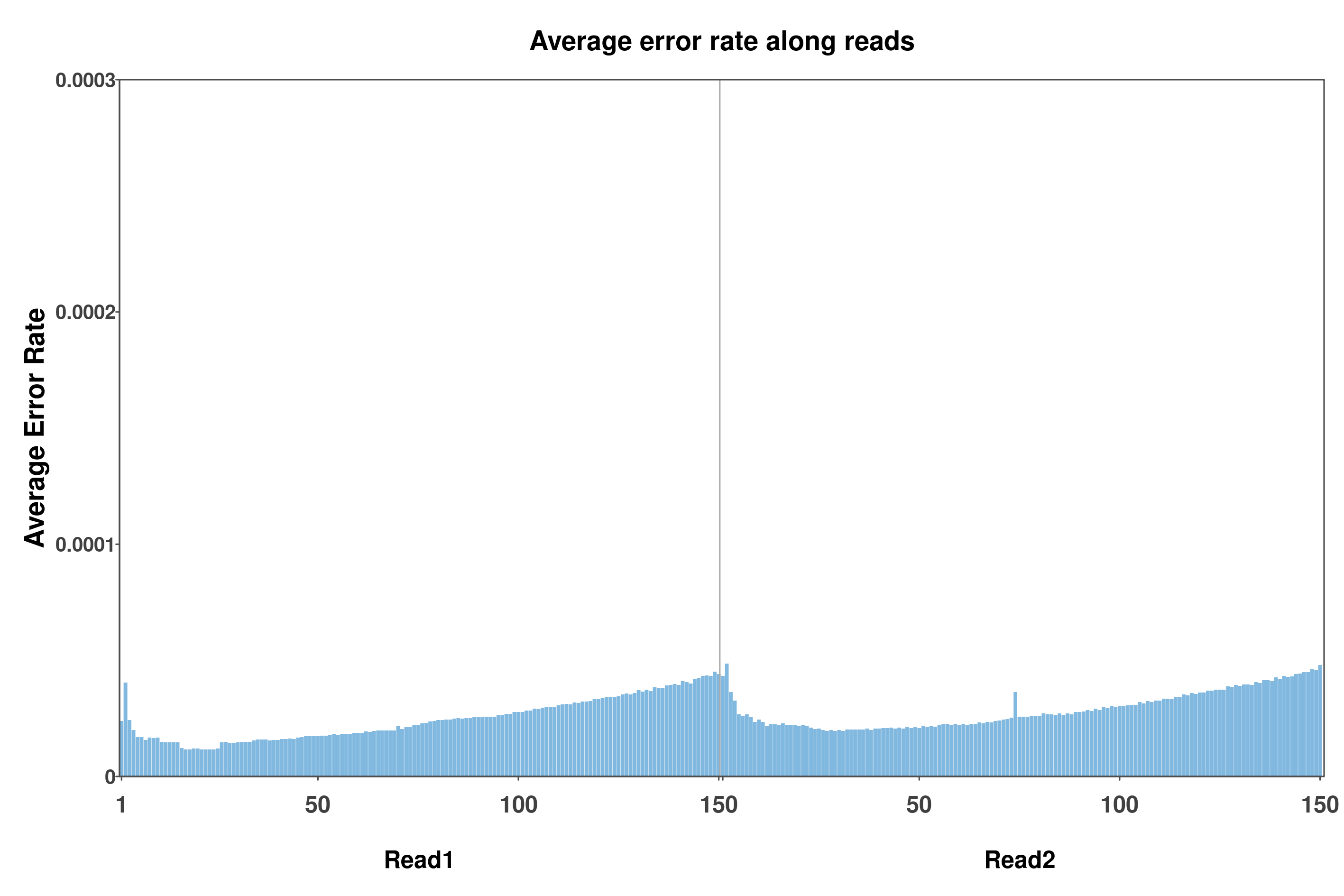

Supplement: Supplementary file 1 [file biology-14-01589-s001.zip › Supplementary Materials S2-Base sequencing error rate analysis/CS-3.quality.png]

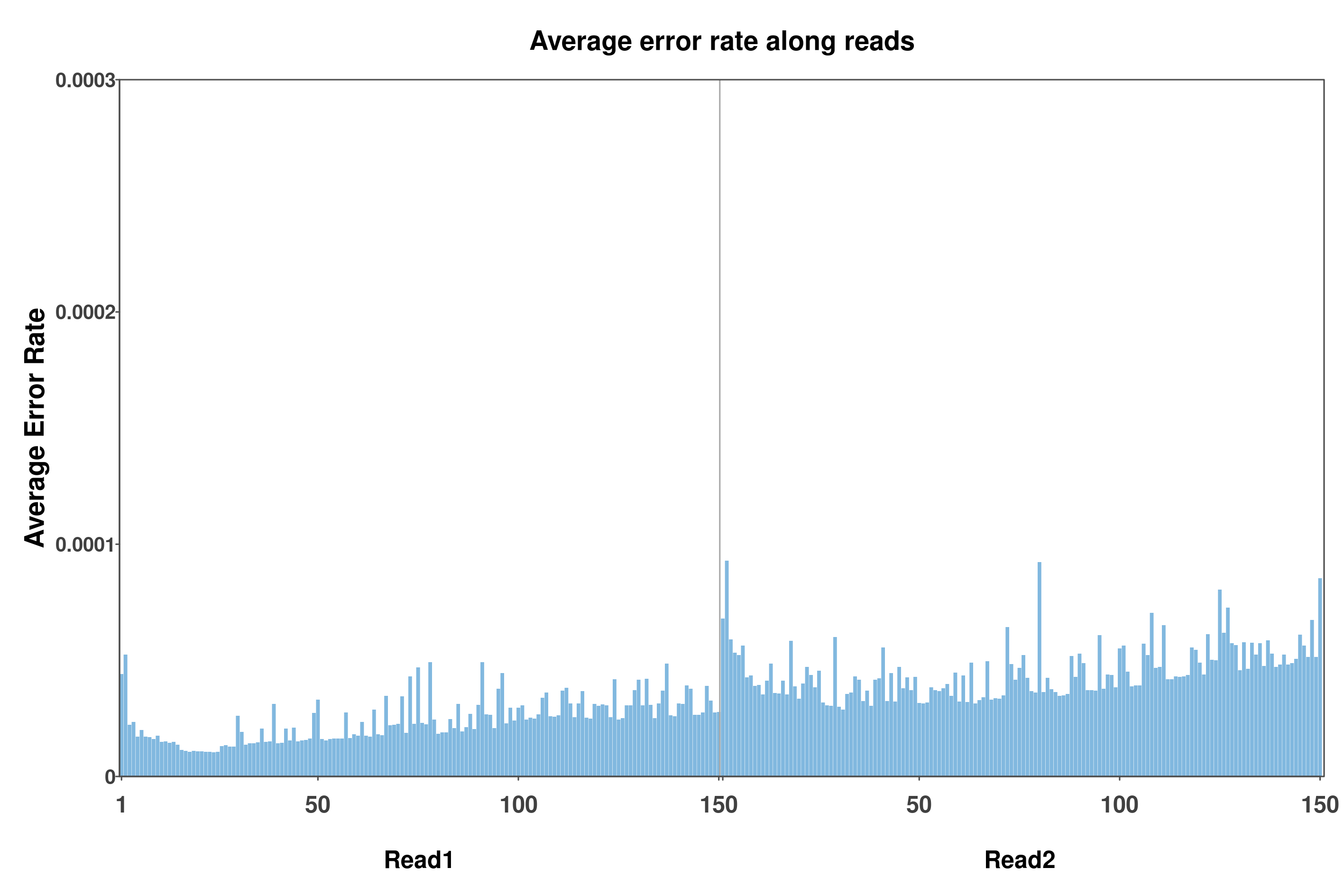

Supplement: Supplementary file 1 [file biology-14-01589-s001.zip › Supplementary Materials S2-Base sequencing error rate analysis/HYS-1.quality.png]

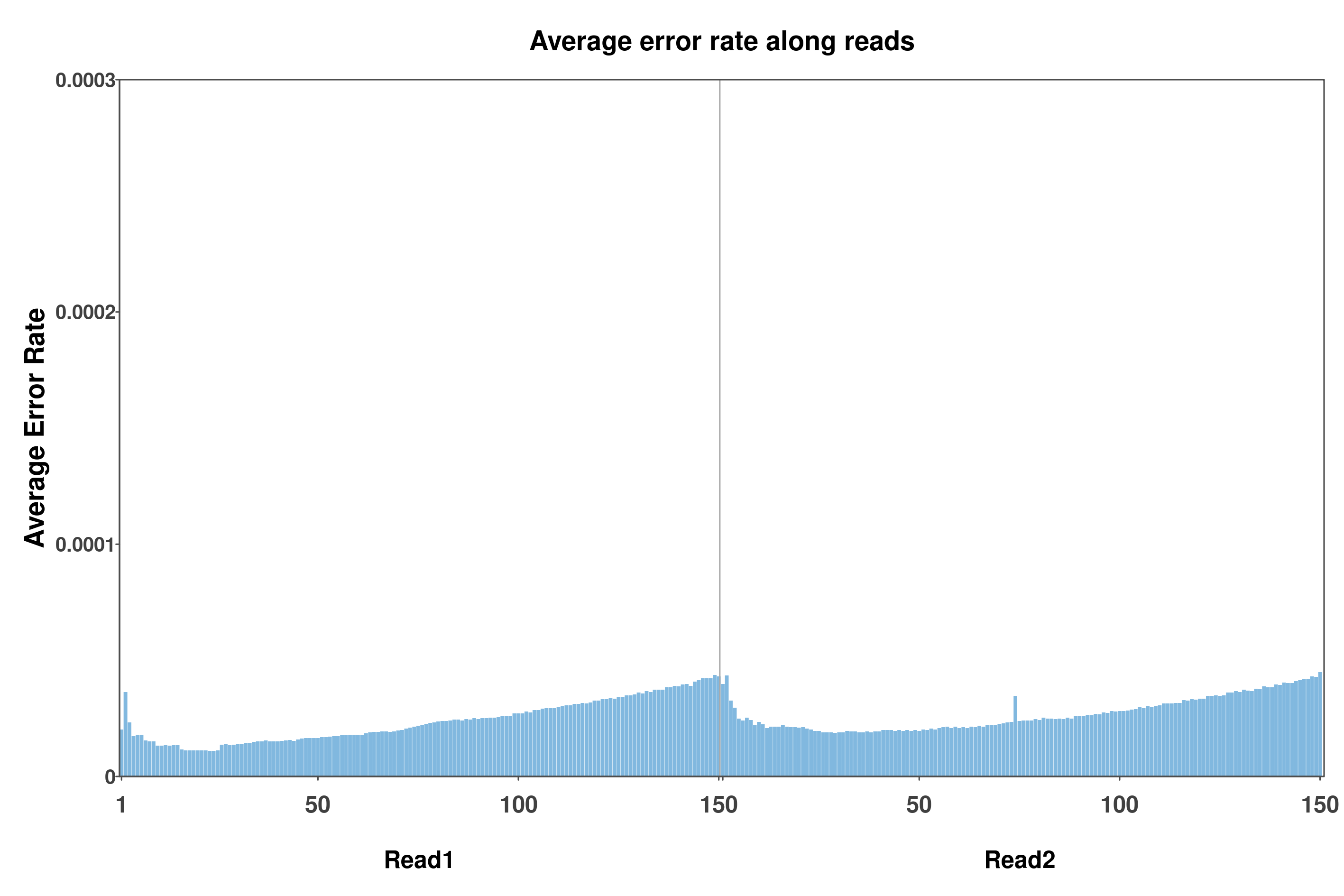

Supplement: Supplementary file 1 [file biology-14-01589-s001.zip › Supplementary Materials S2-Base sequencing error rate analysis/HYS-2.quality.png]

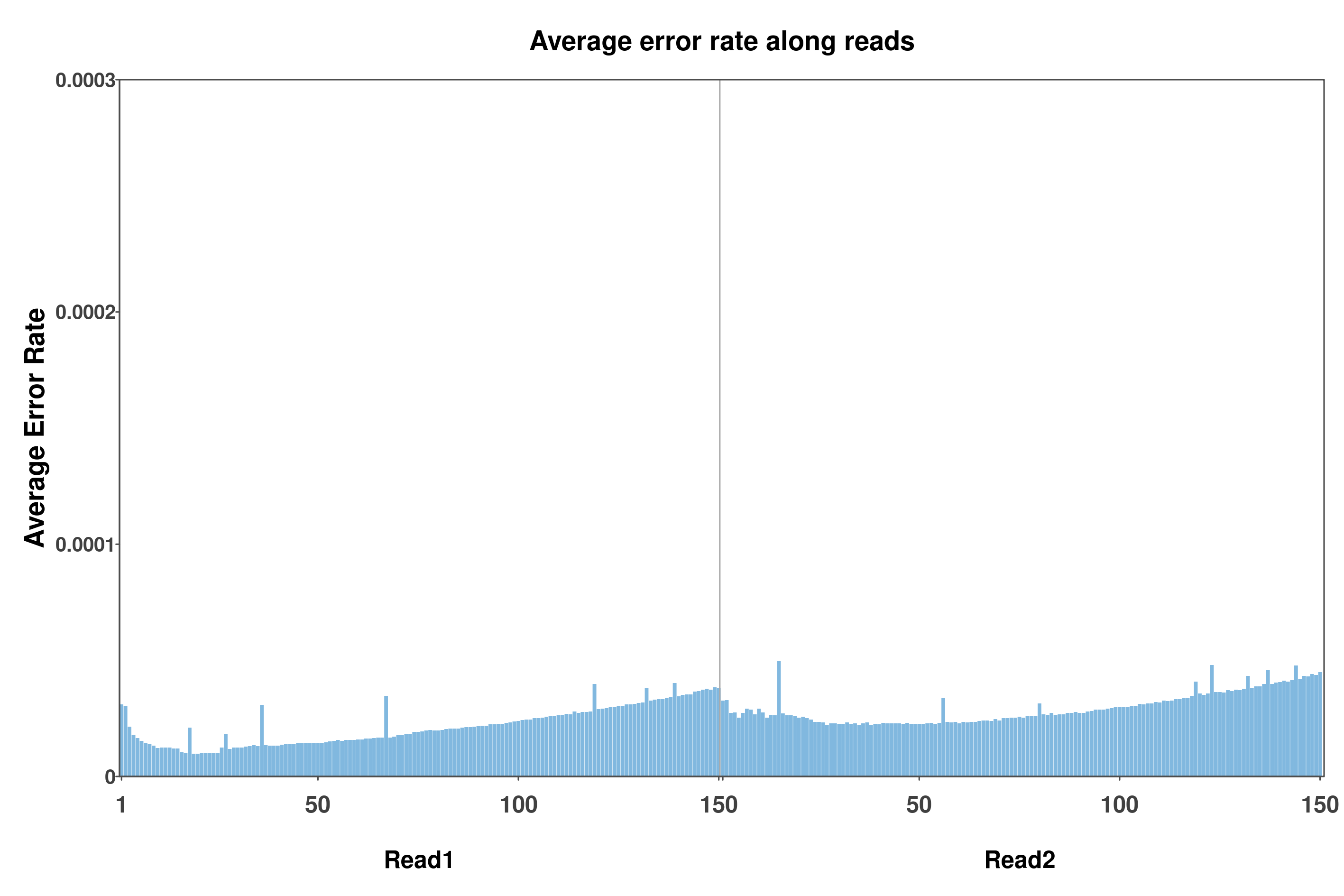

Supplement: Supplementary file 1 [file biology-14-01589-s001.zip › Supplementary Materials S2-Base sequencing error rate analysis/HYS-3.quality.png]

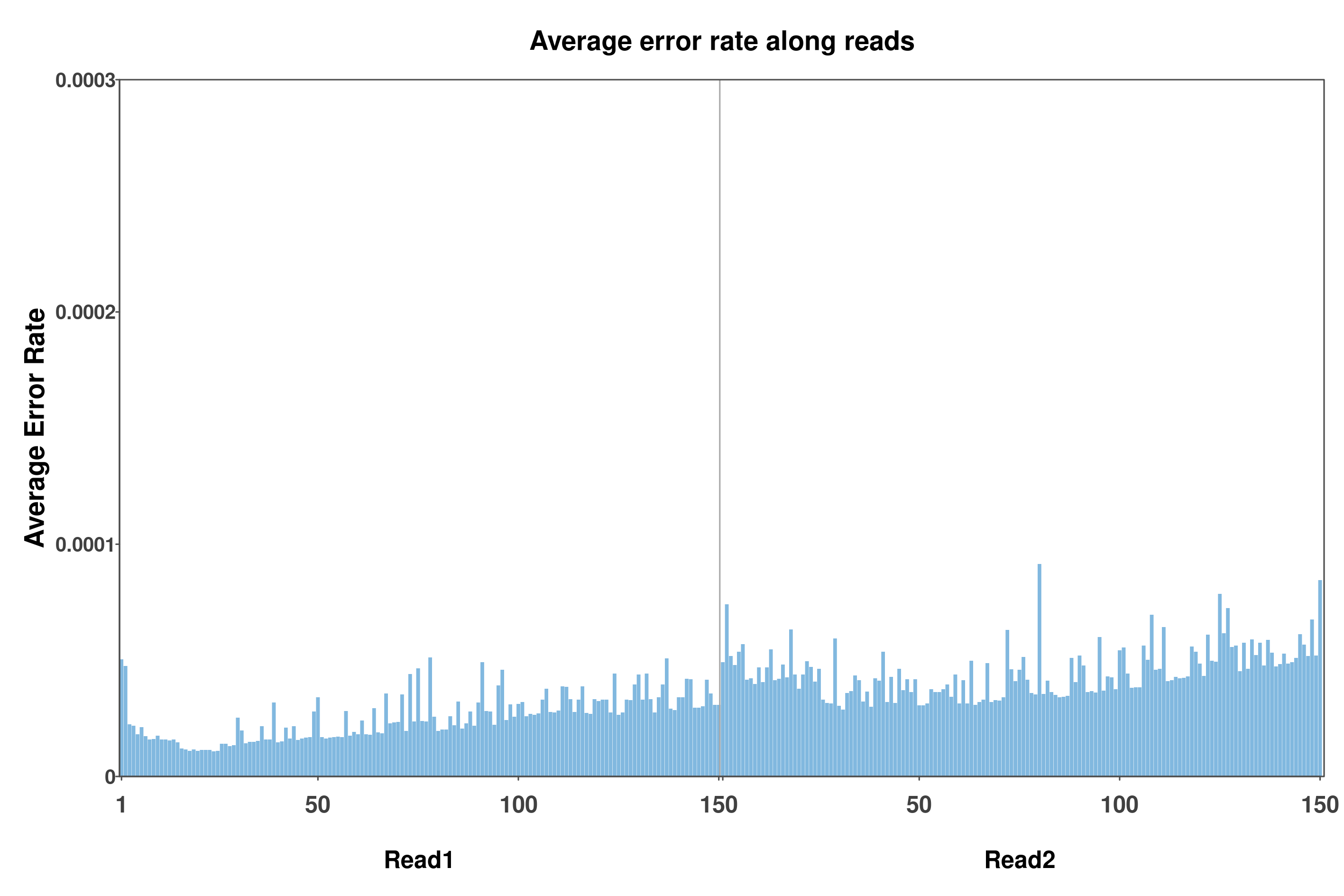

Supplement: Supplementary file 1 [file biology-14-01589-s001.zip › Supplementary Materials S2-Base sequencing error rate analysis/LYS-1.quality.png]

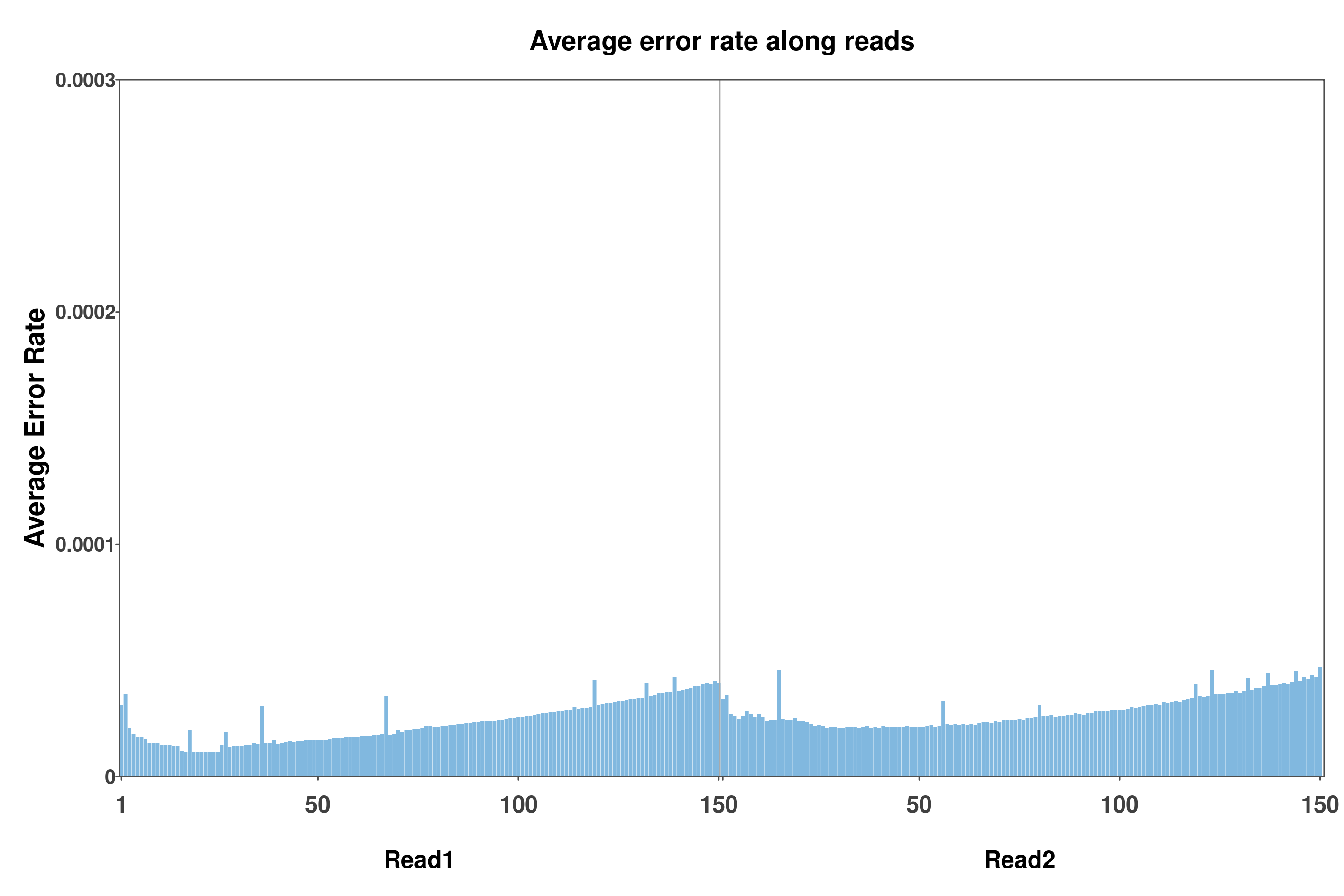

Supplement: Supplementary file 1 [file biology-14-01589-s001.zip › Supplementary Materials S2-Base sequencing error rate analysis/LYS-2.quality.png]

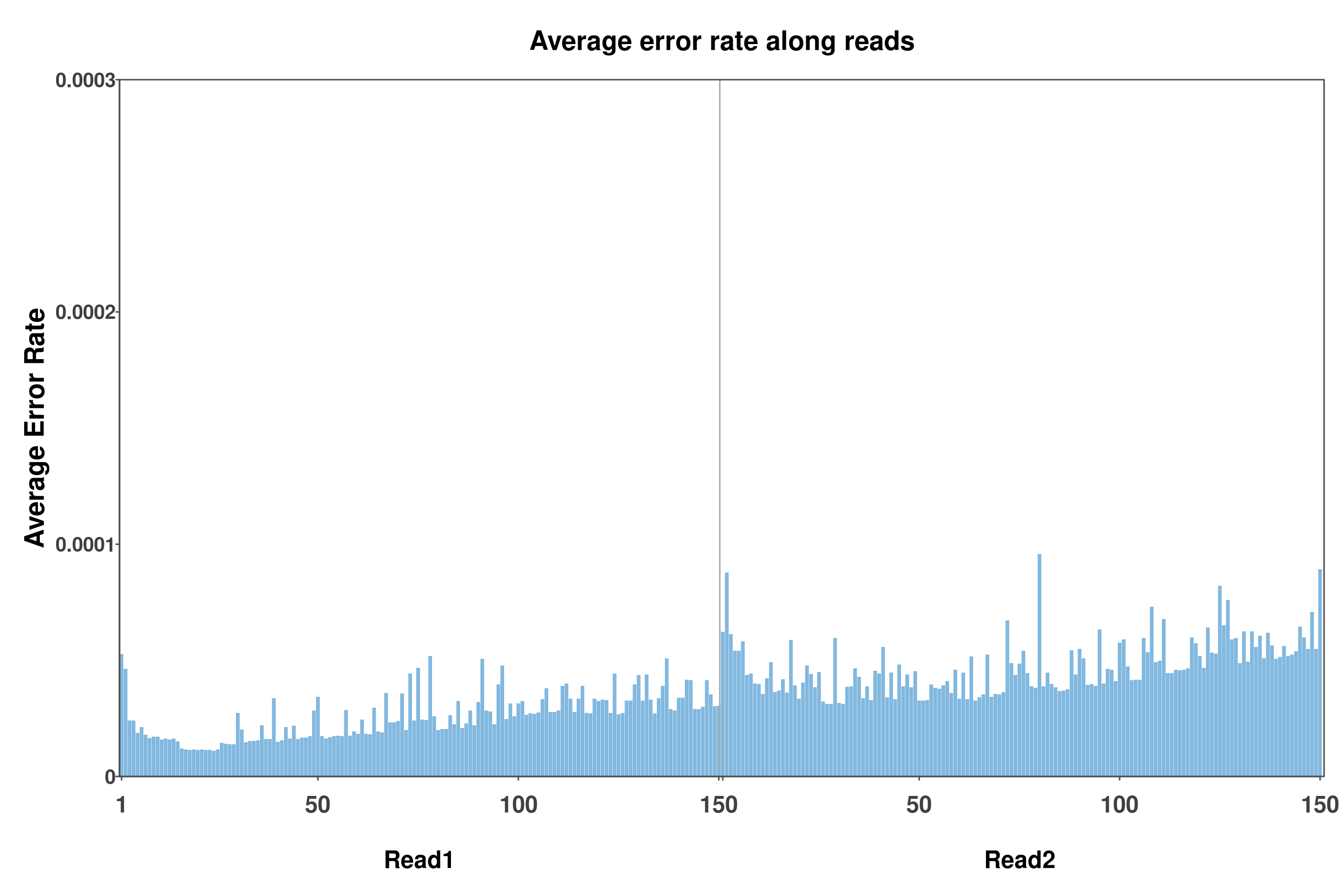

Supplement: Supplementary file 1 [file biology-14-01589-s001.zip › Supplementary Materials S2-Base sequencing error rate analysis/LYS-3.quality.png]

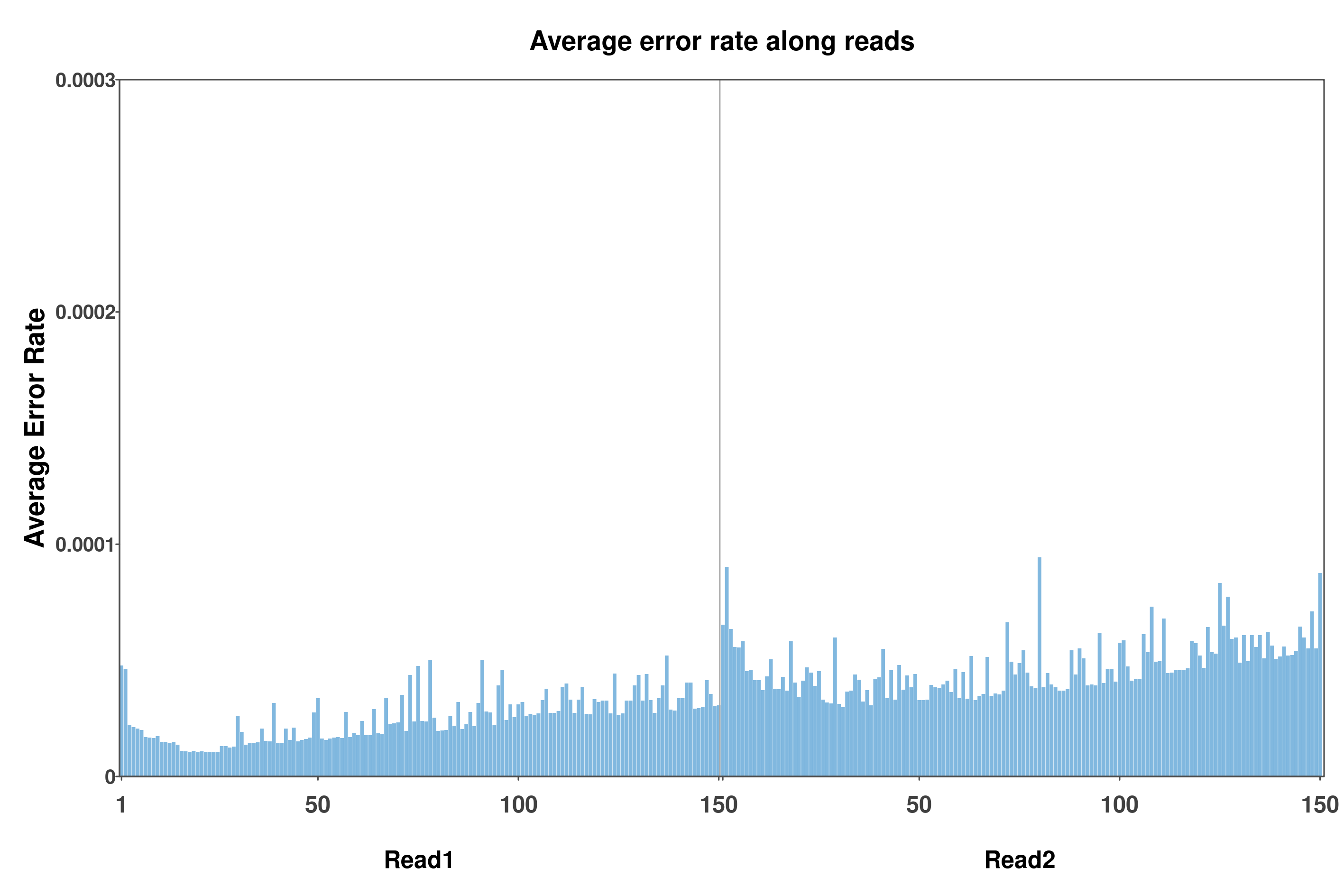

Supplement: Supplementary file 1 [file biology-14-01589-s001.zip › Supplementary Materials S2-Base sequencing error rate analysis/SMS-1.quality.png]

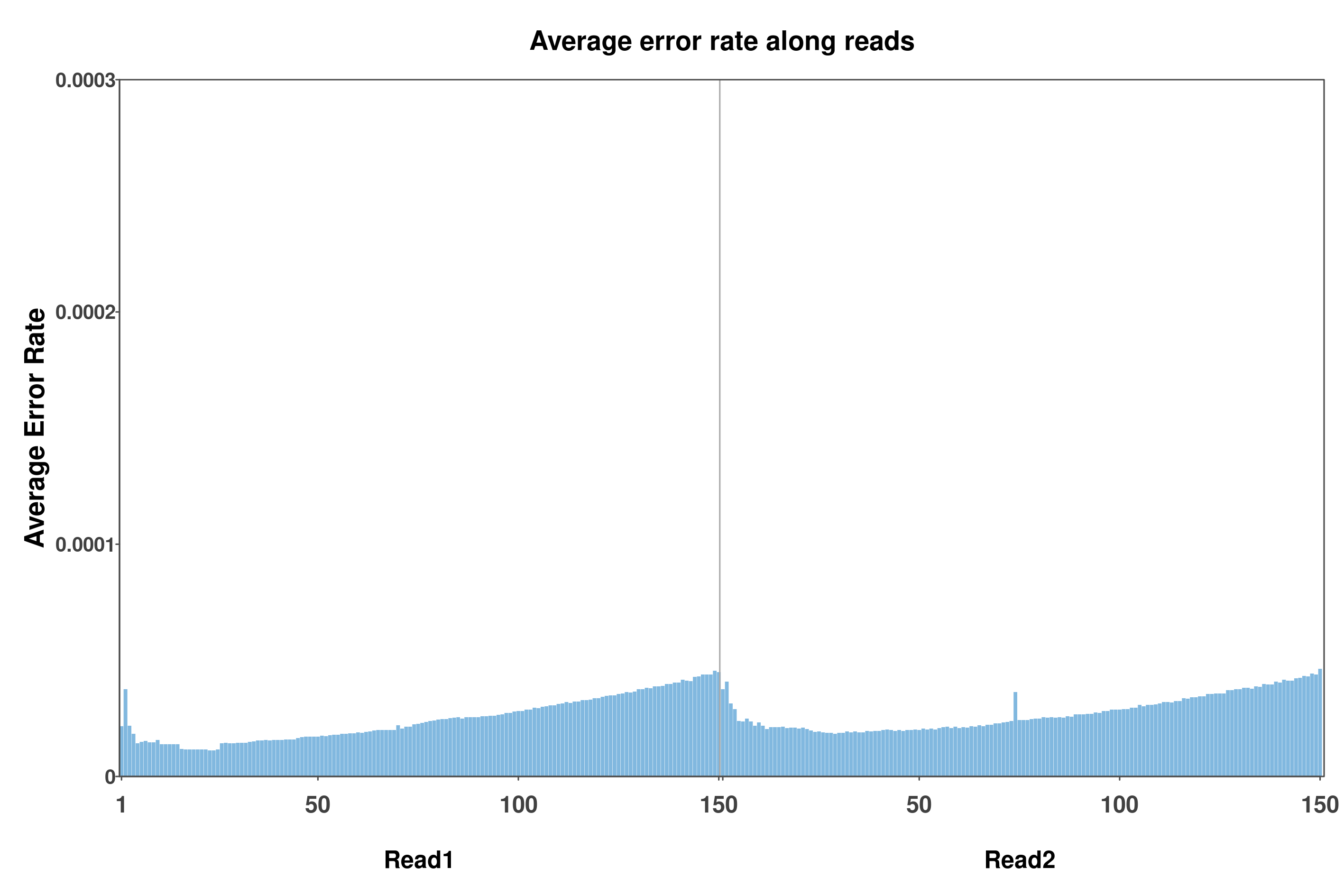

Supplement: Supplementary file 1 [file biology-14-01589-s001.zip › Supplementary Materials S2-Base sequencing error rate analysis/SMS-2.quality.png]

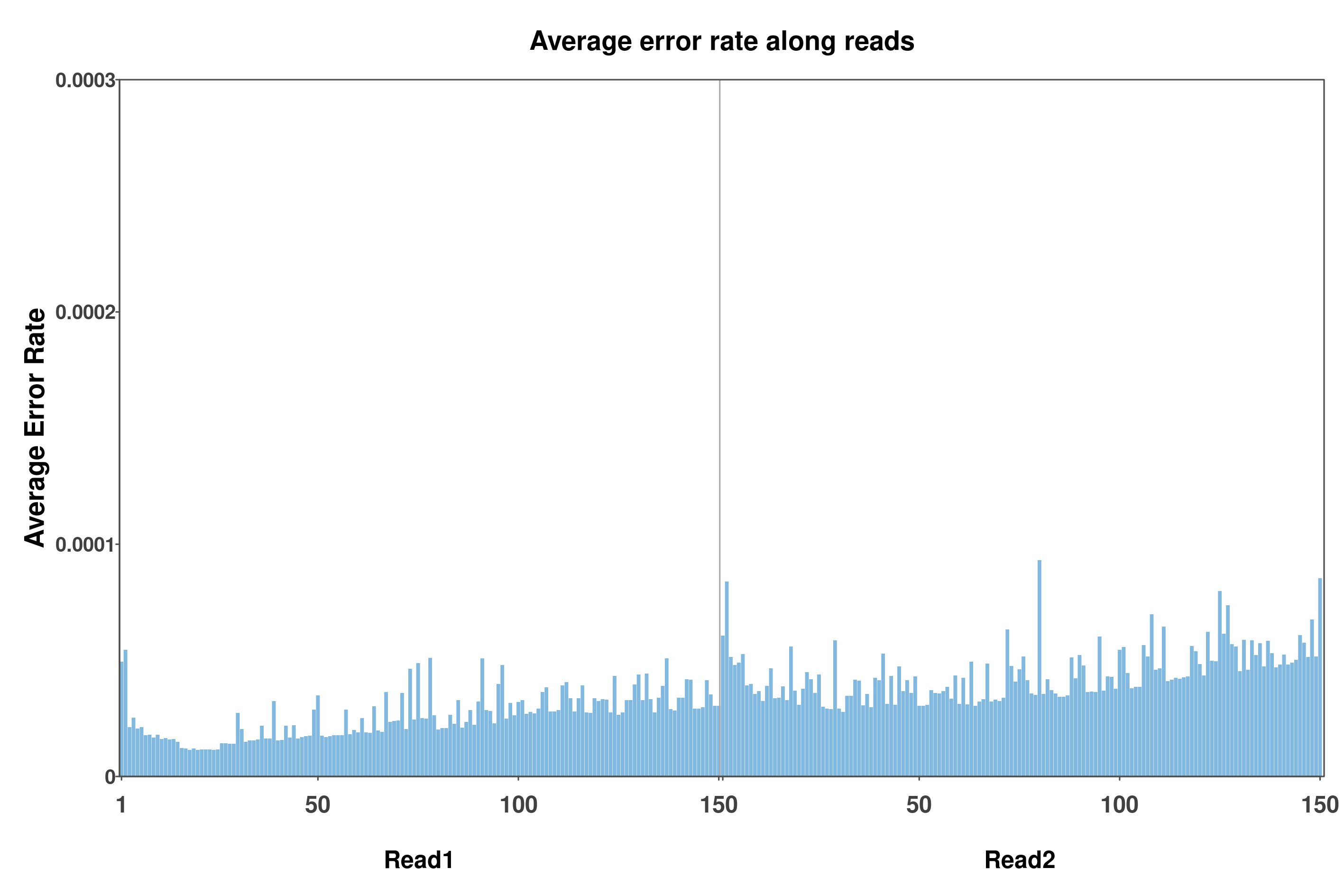

Supplement: Supplementary file 1 [file biology-14-01589-s001.zip › Supplementary Materials S2-Base sequencing error rate analysis/SMS-3.quality.png]

Base Distribution

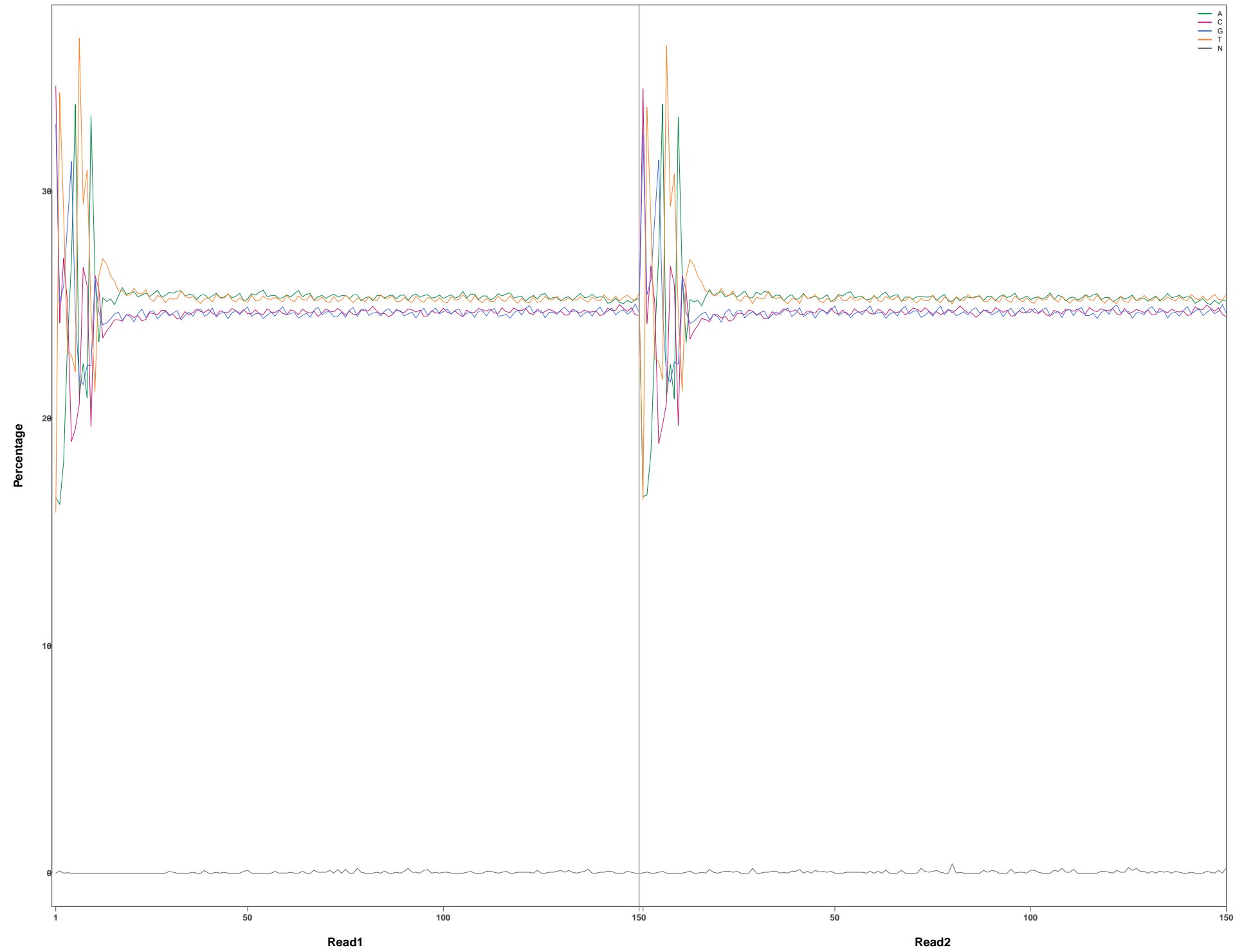

Supplement: Supplementary file 1 [file biology-14-01589-s001.zip › Supplementary Materials S3-the samples remained stable during the sequencing process/CS-1.acgtn.pdf]

Base Distribution

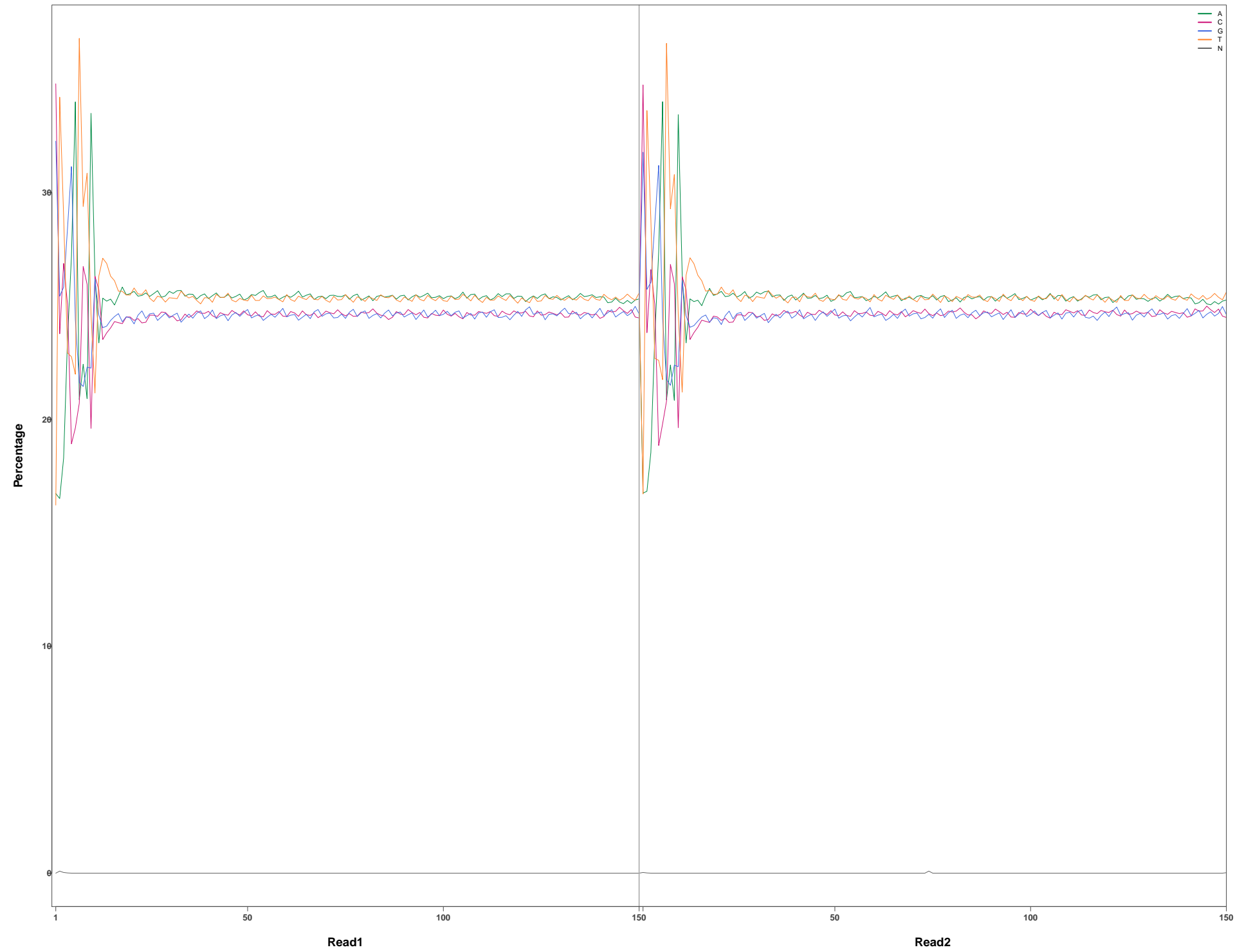

Supplement: Supplementary file 1 [file biology-14-01589-s001.zip › Supplementary Materials S3-the samples remained stable during the sequencing process/CS-2.acgtn.pdf]

Base Distribution

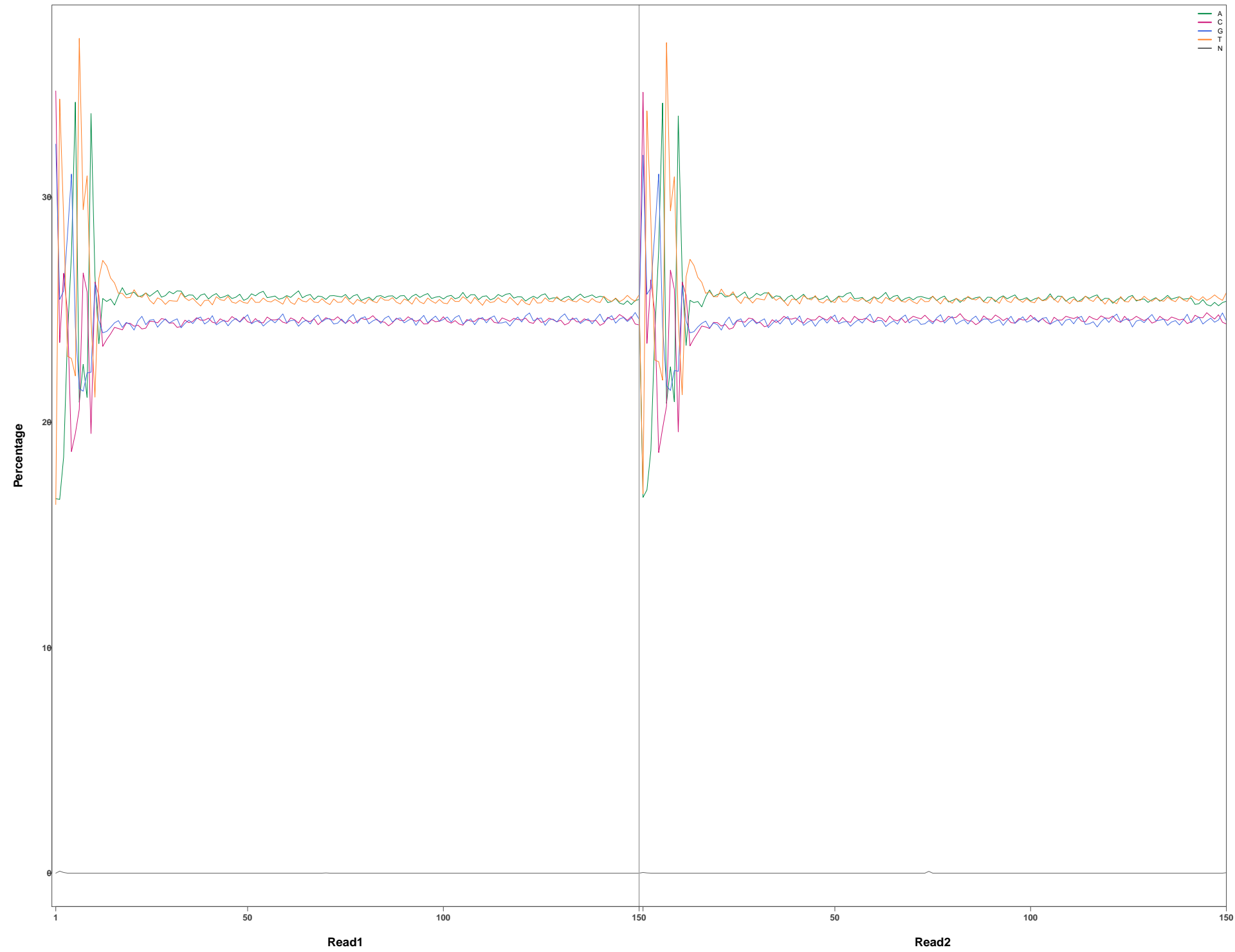

Supplement: Supplementary file 1 [file biology-14-01589-s001.zip › Supplementary Materials S3-the samples remained stable during the sequencing process/CS-3.acgtn.pdf]

Base Distribution

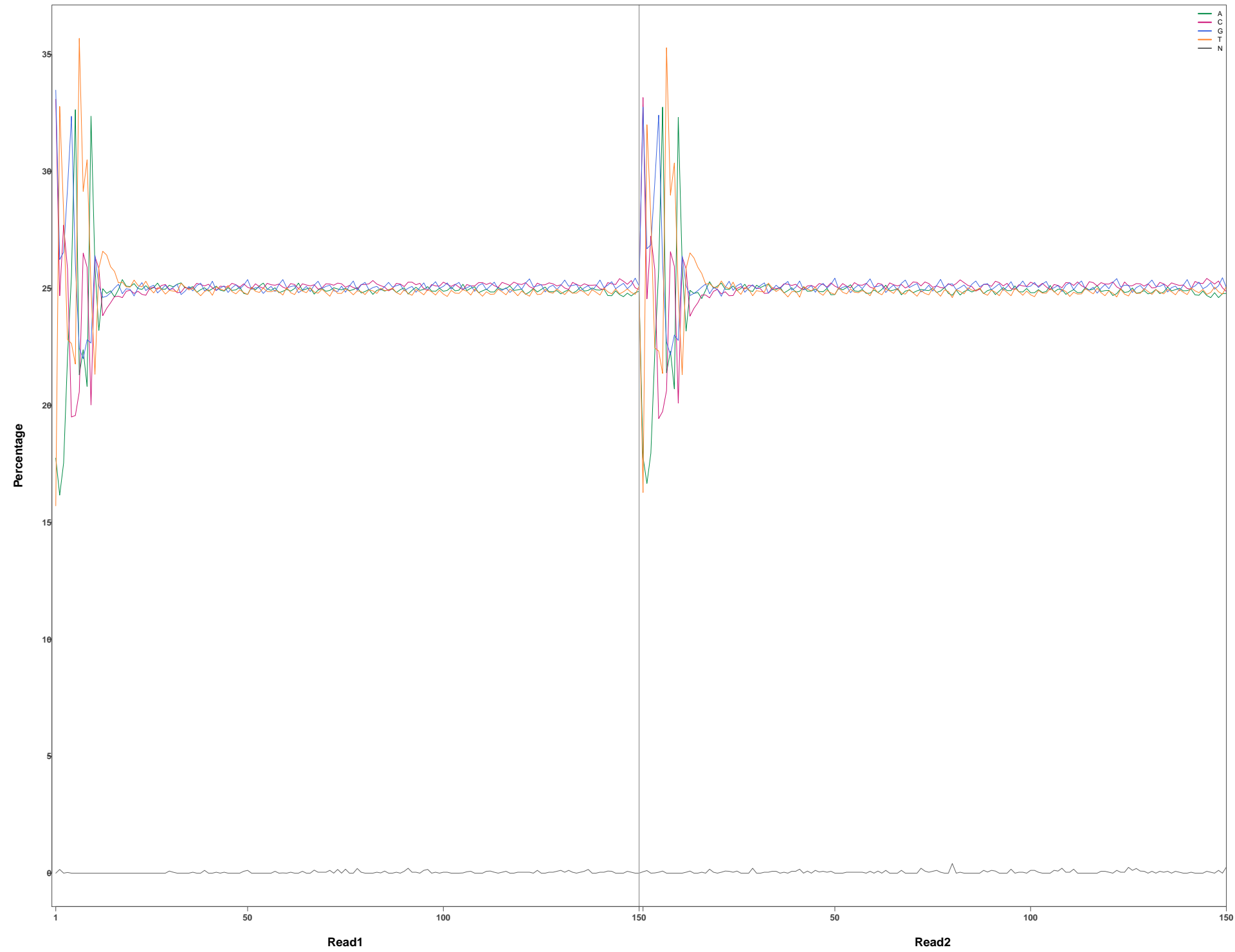

Supplement: Supplementary file 1 [file biology-14-01589-s001.zip › Supplementary Materials S3-the samples remained stable during the sequencing process/HYS-1.acgtn.pdf]

Base Distribution

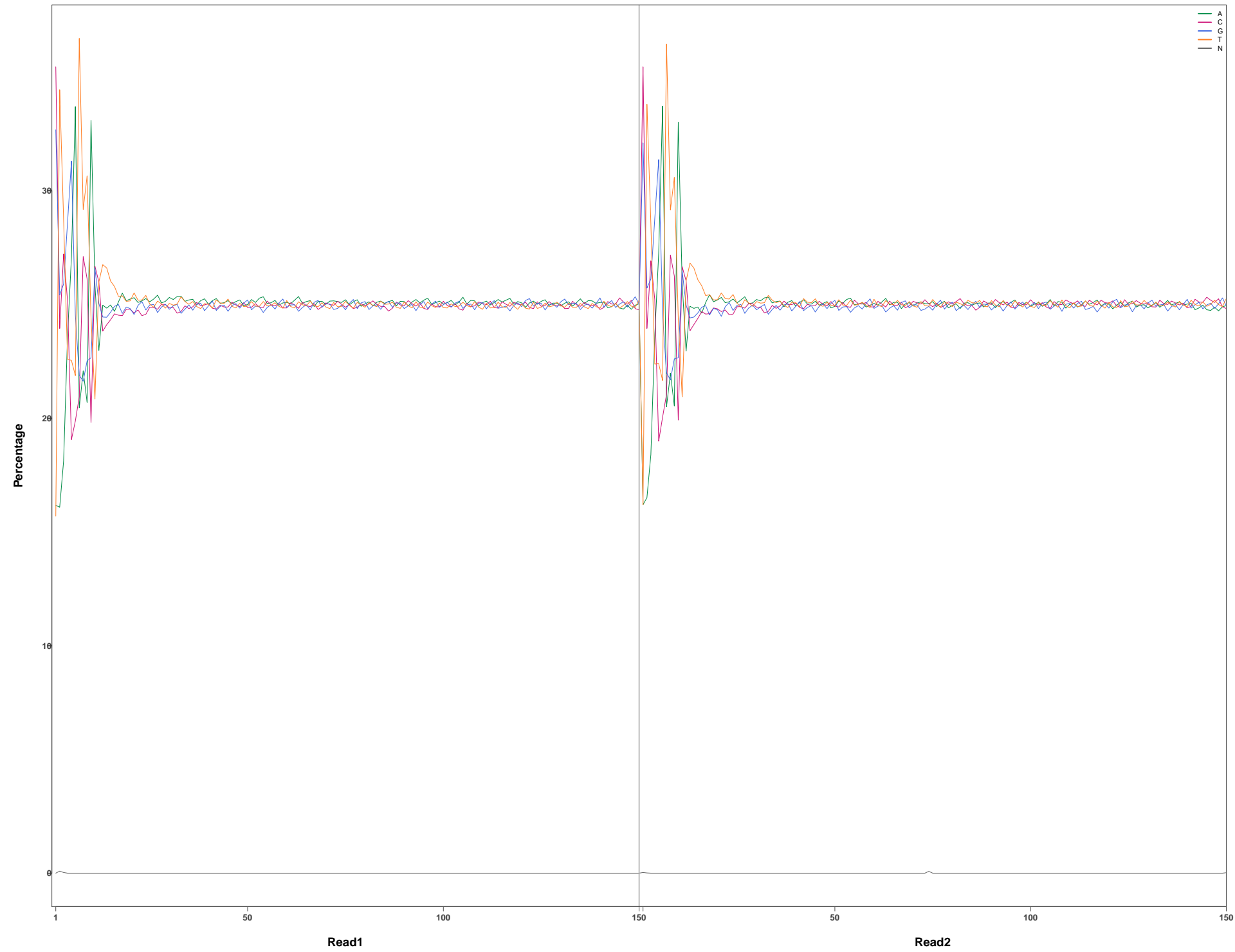

Supplement: Supplementary file 1 [file biology-14-01589-s001.zip › Supplementary Materials S3-the samples remained stable during the sequencing process/HYS-2.acgtn.pdf]

Base Distribution

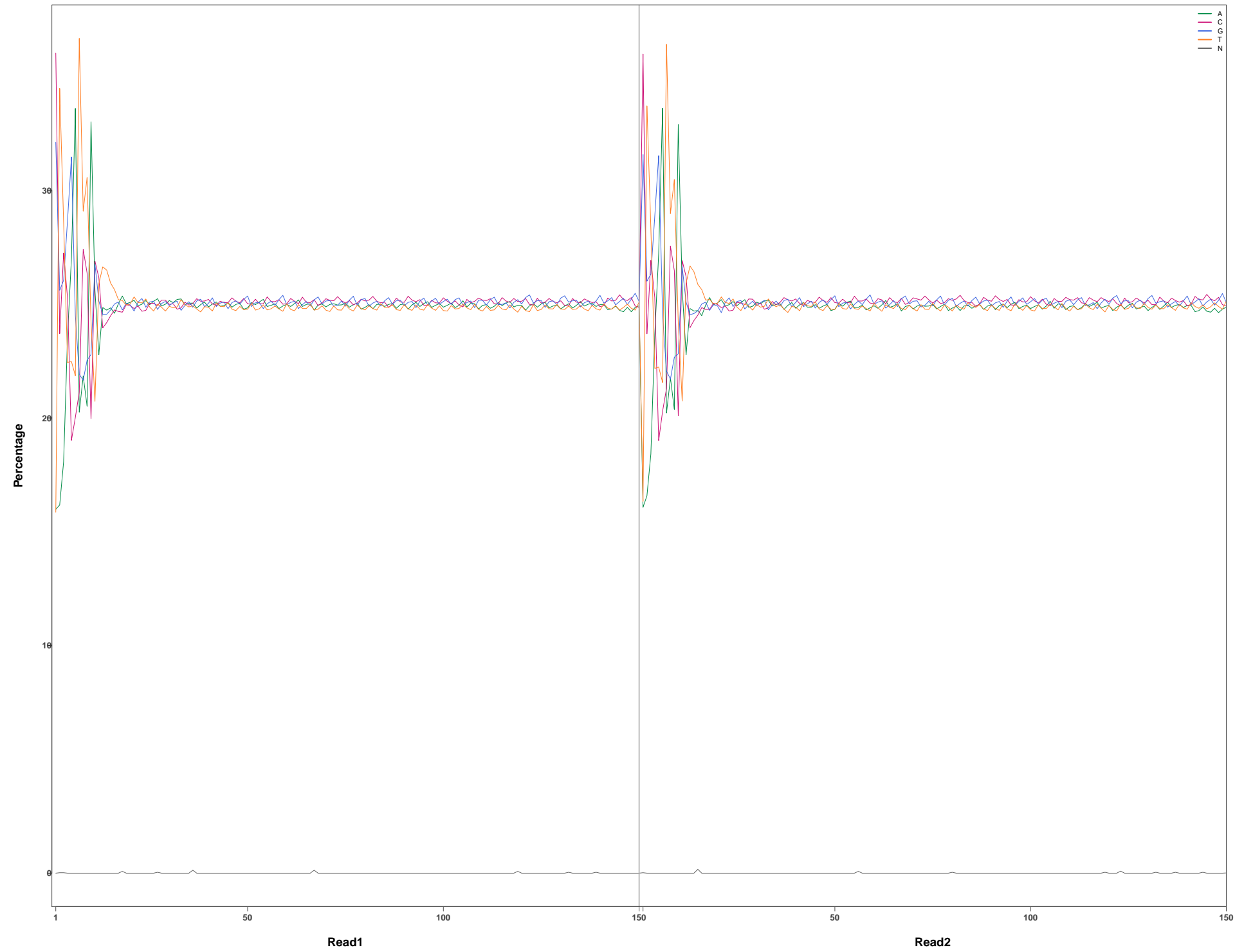

Supplement: Supplementary file 1 [file biology-14-01589-s001.zip › Supplementary Materials S3-the samples remained stable during the sequencing process/HYS-3.acgtn.pdf]

Base Distribution

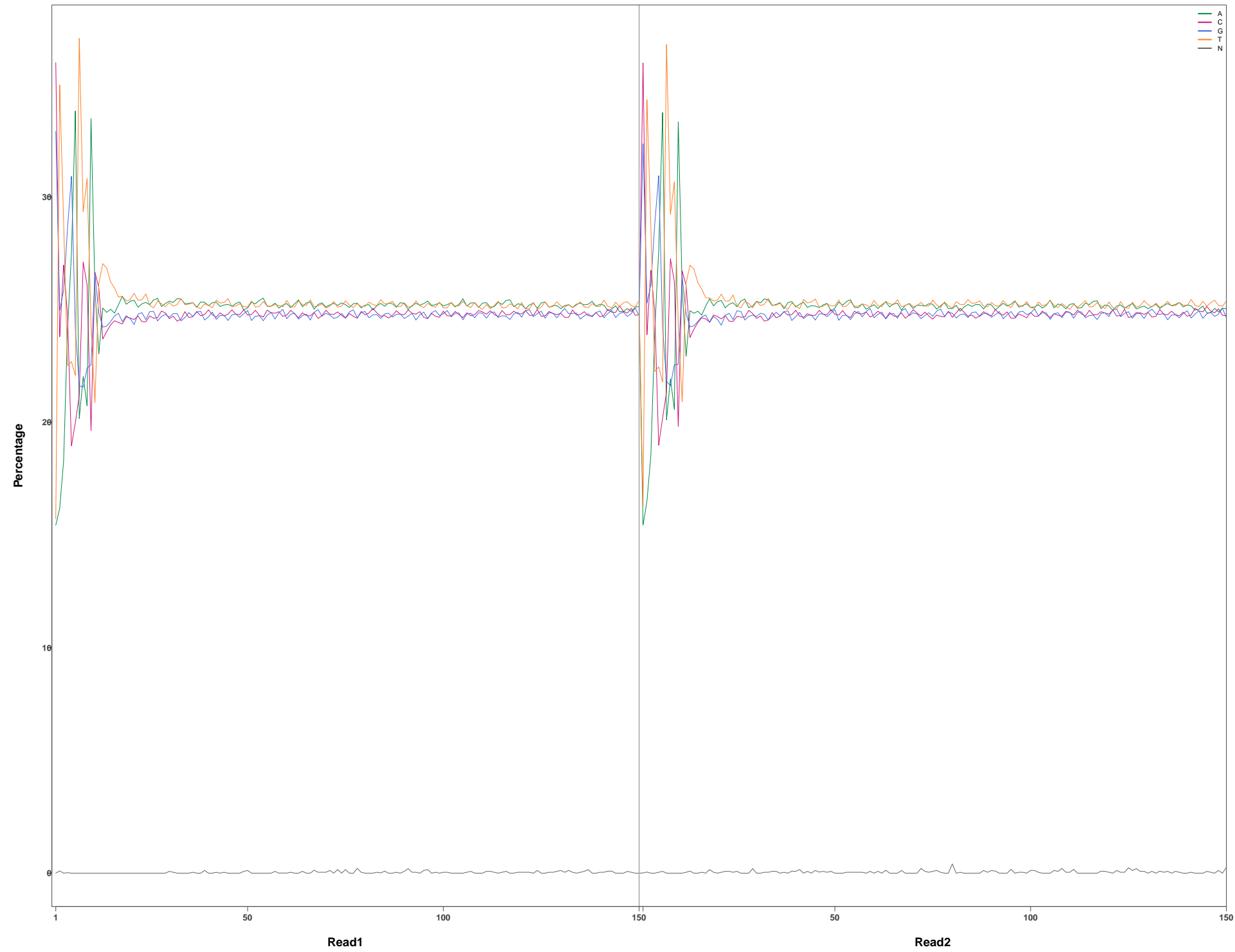

Supplement: Supplementary file 1 [file biology-14-01589-s001.zip › Supplementary Materials S3-the samples remained stable during the sequencing process/LYS-1.acgtn.pdf]

Base Distribution

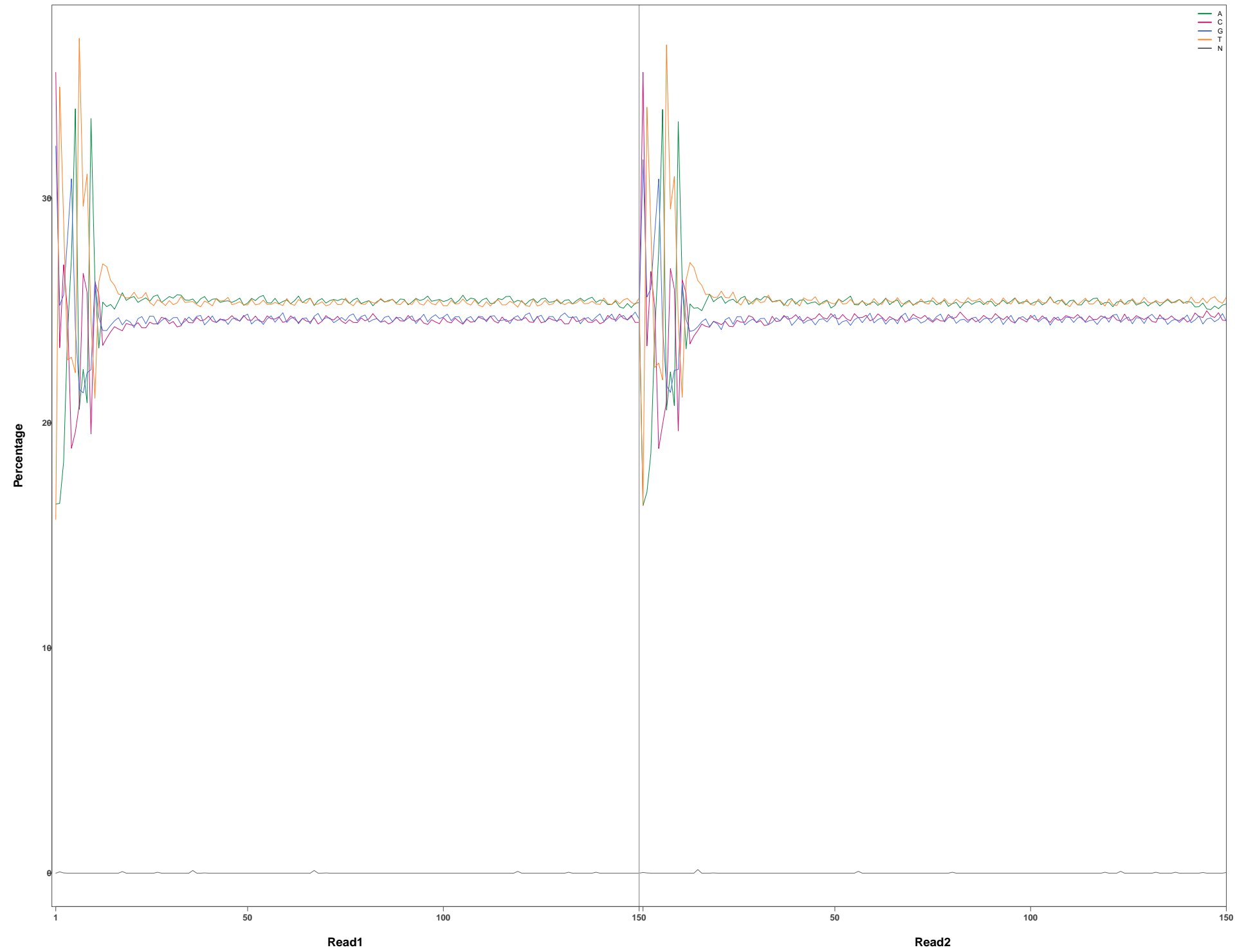

Supplement: Supplementary file 1 [file biology-14-01589-s001.zip › Supplementary Materials S3-the samples remained stable during the sequencing process/LYS-2.acgtn.pdf]

Base Distribution

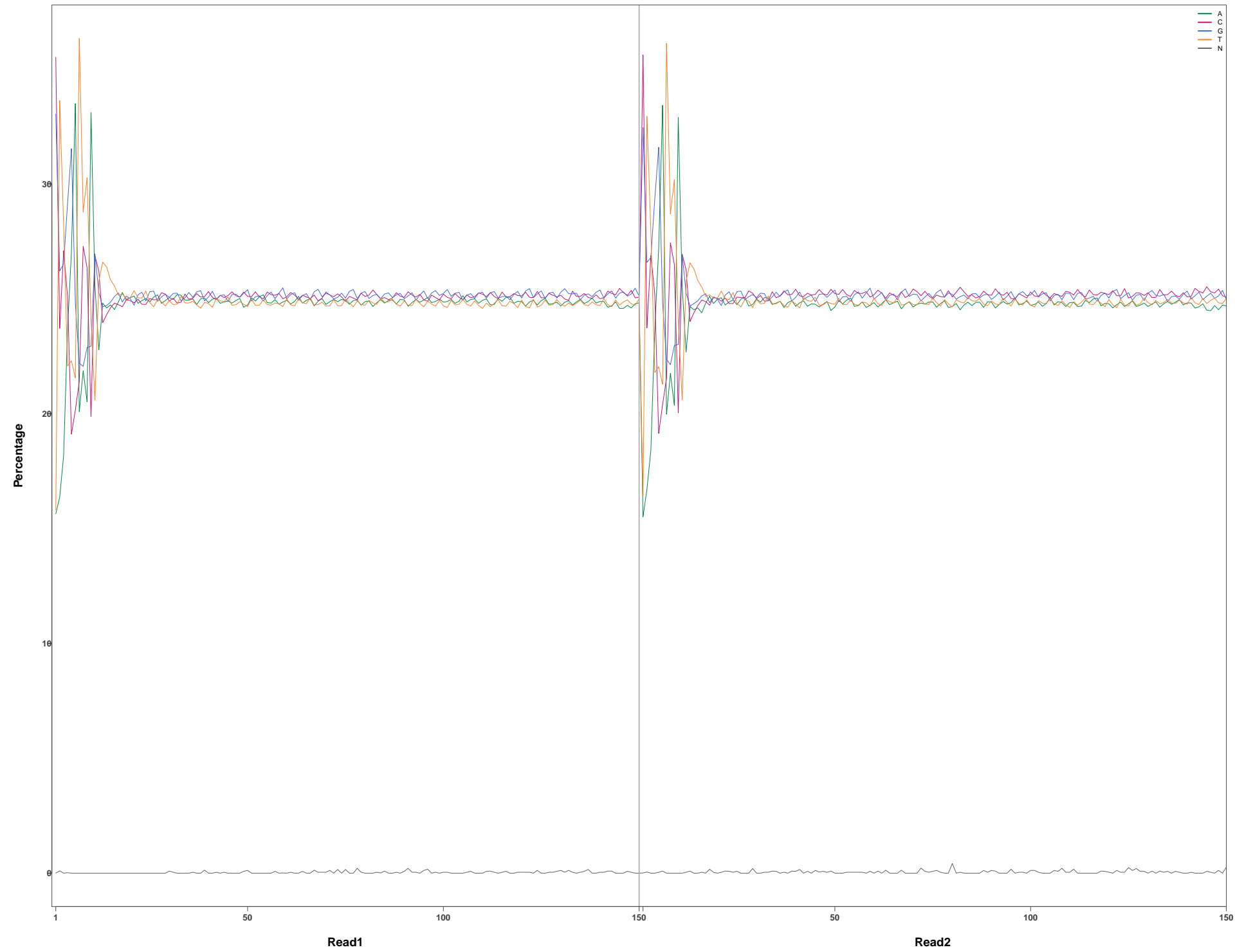

Supplement: Supplementary file 1 [file biology-14-01589-s001.zip › Supplementary Materials S3-the samples remained stable during the sequencing process/LYS-3.acgtn.pdf]

Base Distribution

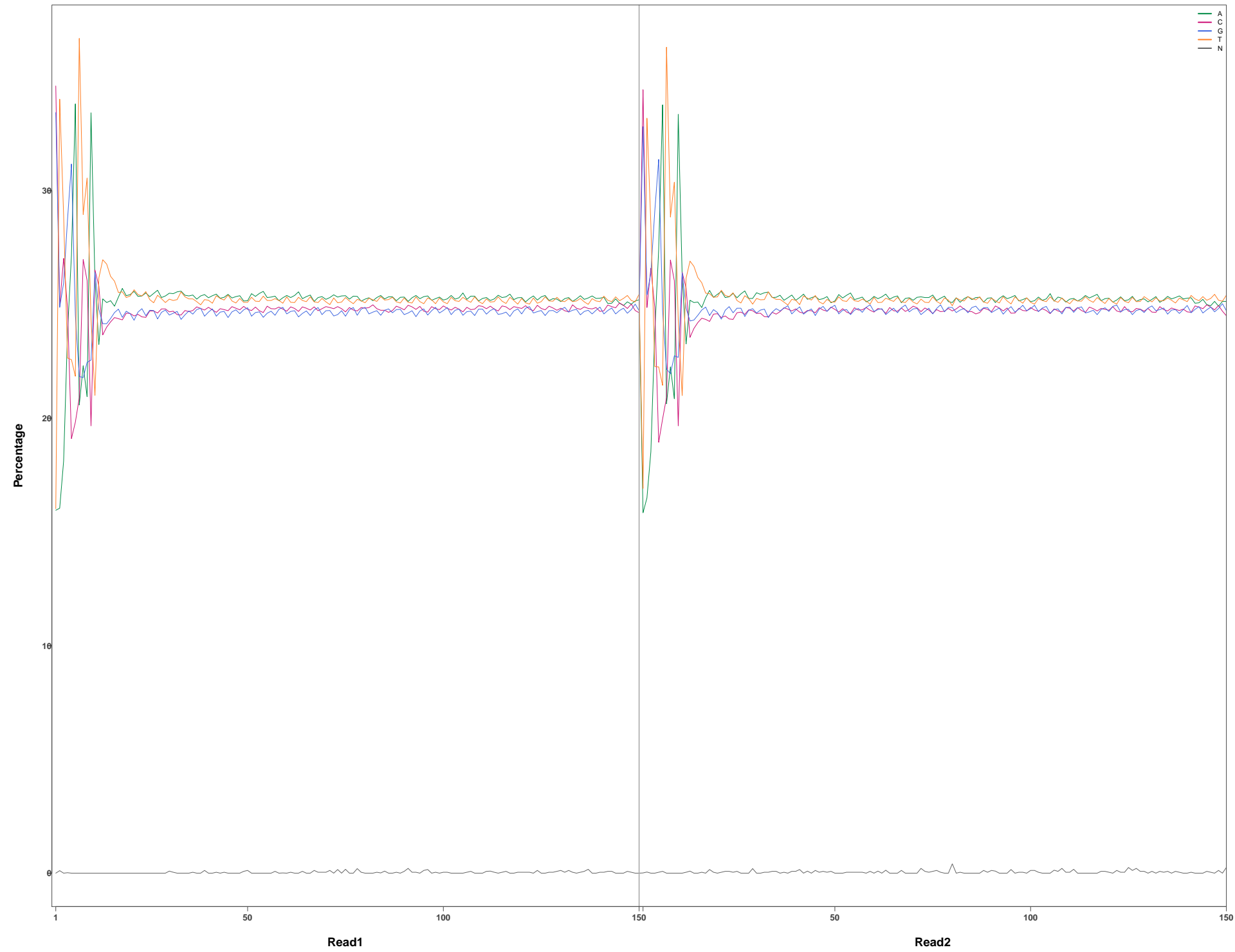

Supplement: Supplementary file 1 [file biology-14-01589-s001.zip › Supplementary Materials S3-the samples remained stable during the sequencing process/SMS-1.acgtn.pdf]

Base Distribution

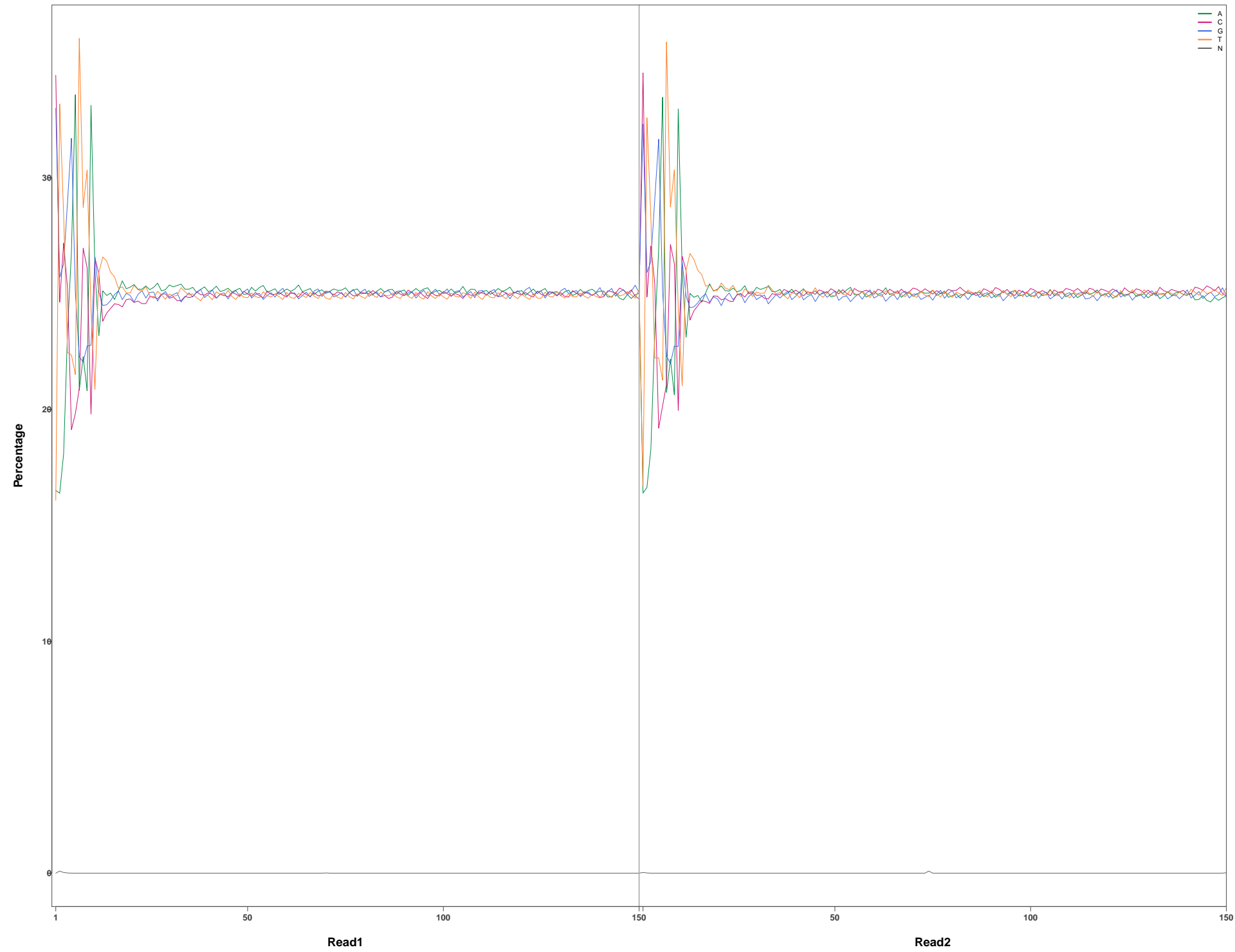

Supplement: Supplementary file 1 [file biology-14-01589-s001.zip › Supplementary Materials S3-the samples remained stable during the sequencing process/SMS-2.acgtn.pdf]

Base Distribution

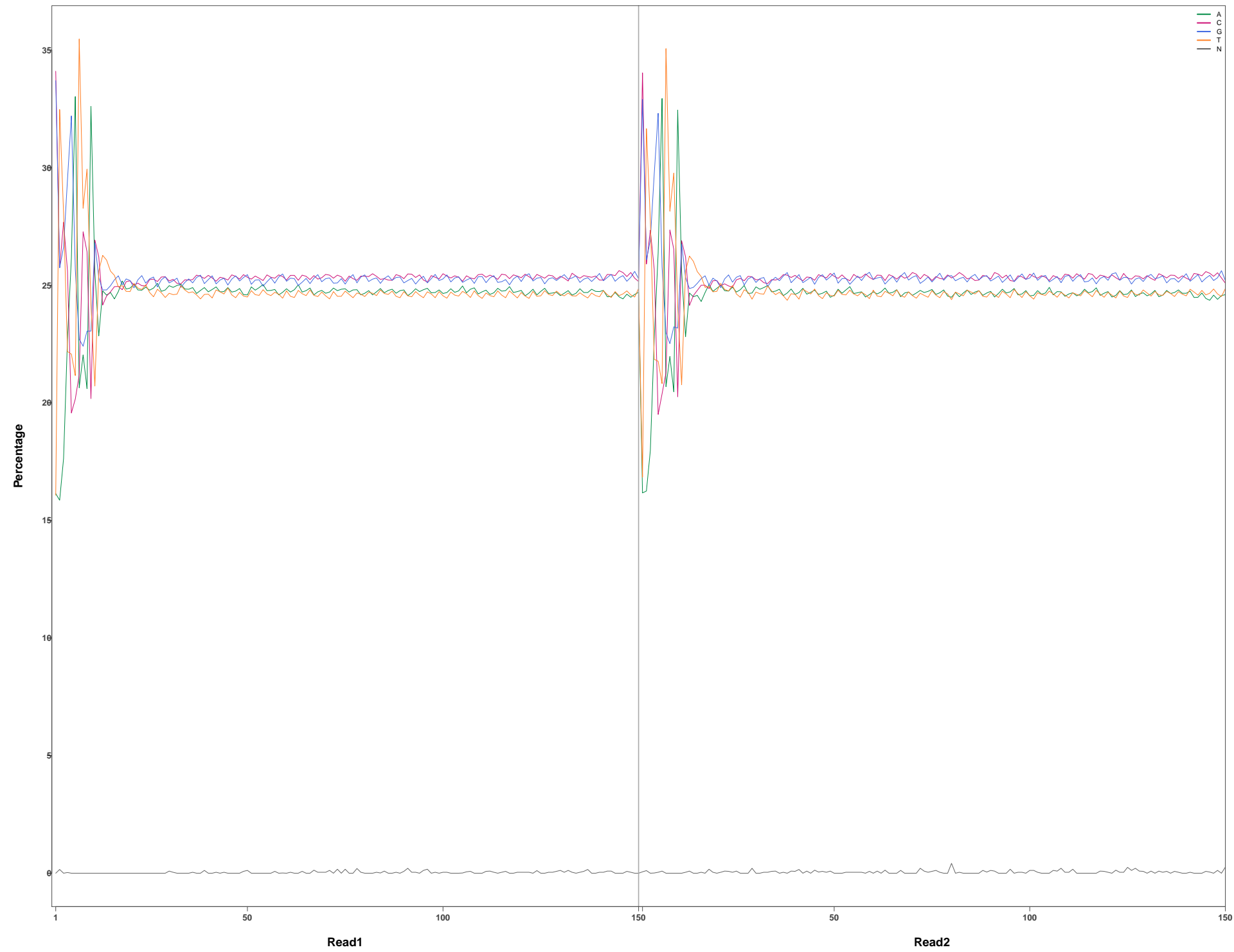

Supplement: Supplementary file 1 [file biology-14-01589-s001.zip › Supplementary Materials S3-the samples remained stable during the sequencing process/SMS-3.acgtn.pdf]

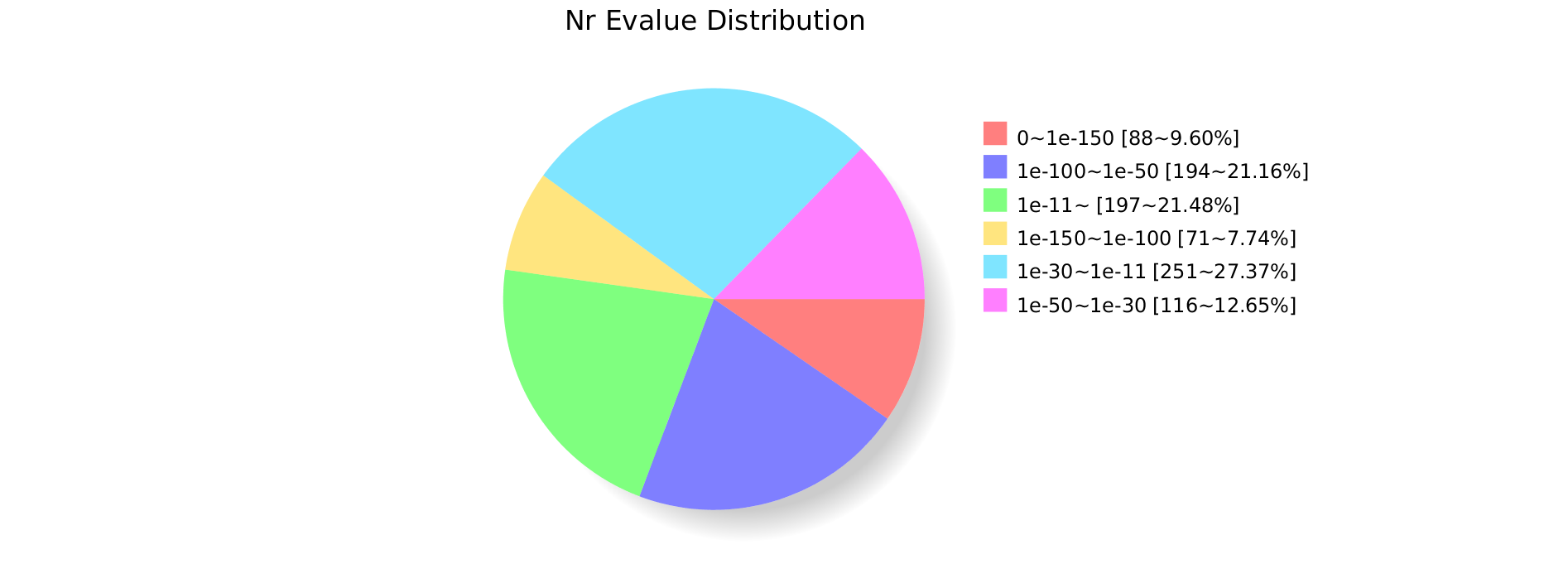

Supplement: Supplementary file 1 [file biology-14-01589-s001.zip › Supplementary Materials S8-The novel genes were sequentially aligned with the databases and were annotated/Sus_scrofa.newGene.longest_transcript.pep.fa.nr.evalue.png]

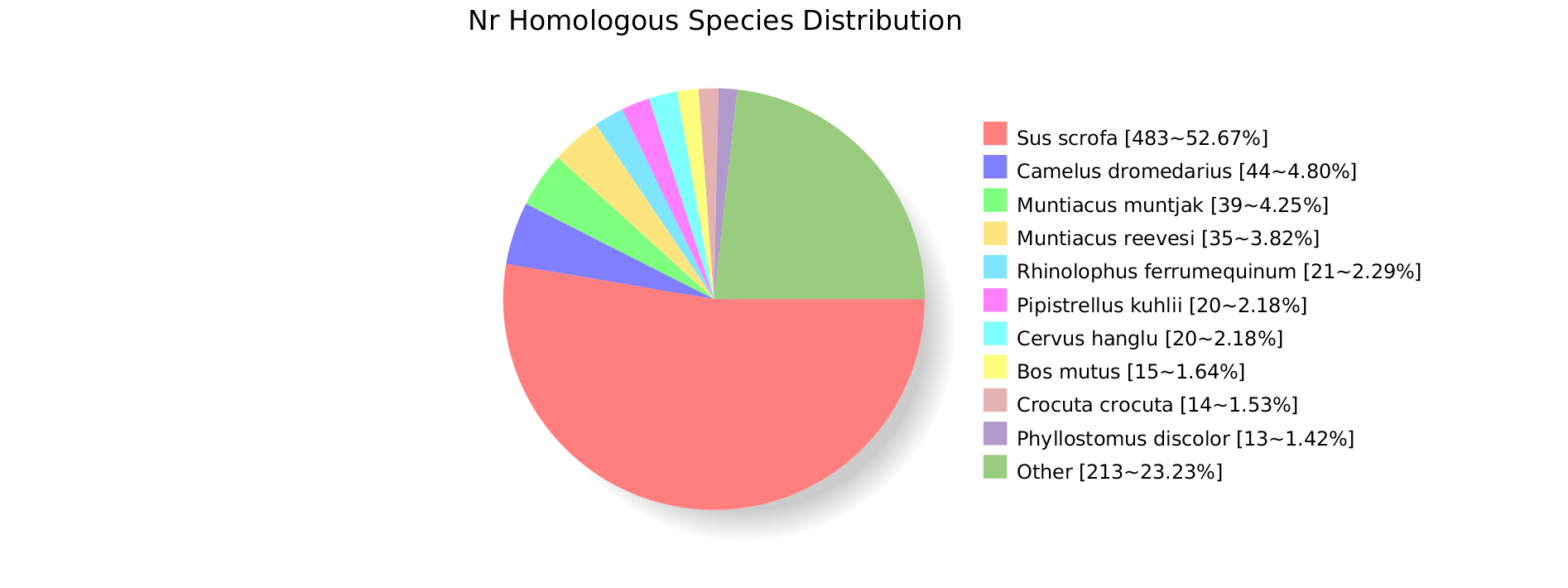

Supplement: Supplementary file 1 [file biology-14-01589-s001.zip › Supplementary Materials S8-The novel genes were sequentially aligned with the databases and were annotated/Sus_scrofa.newGene.longest_transcript.pep.fa.nr.lib.png]

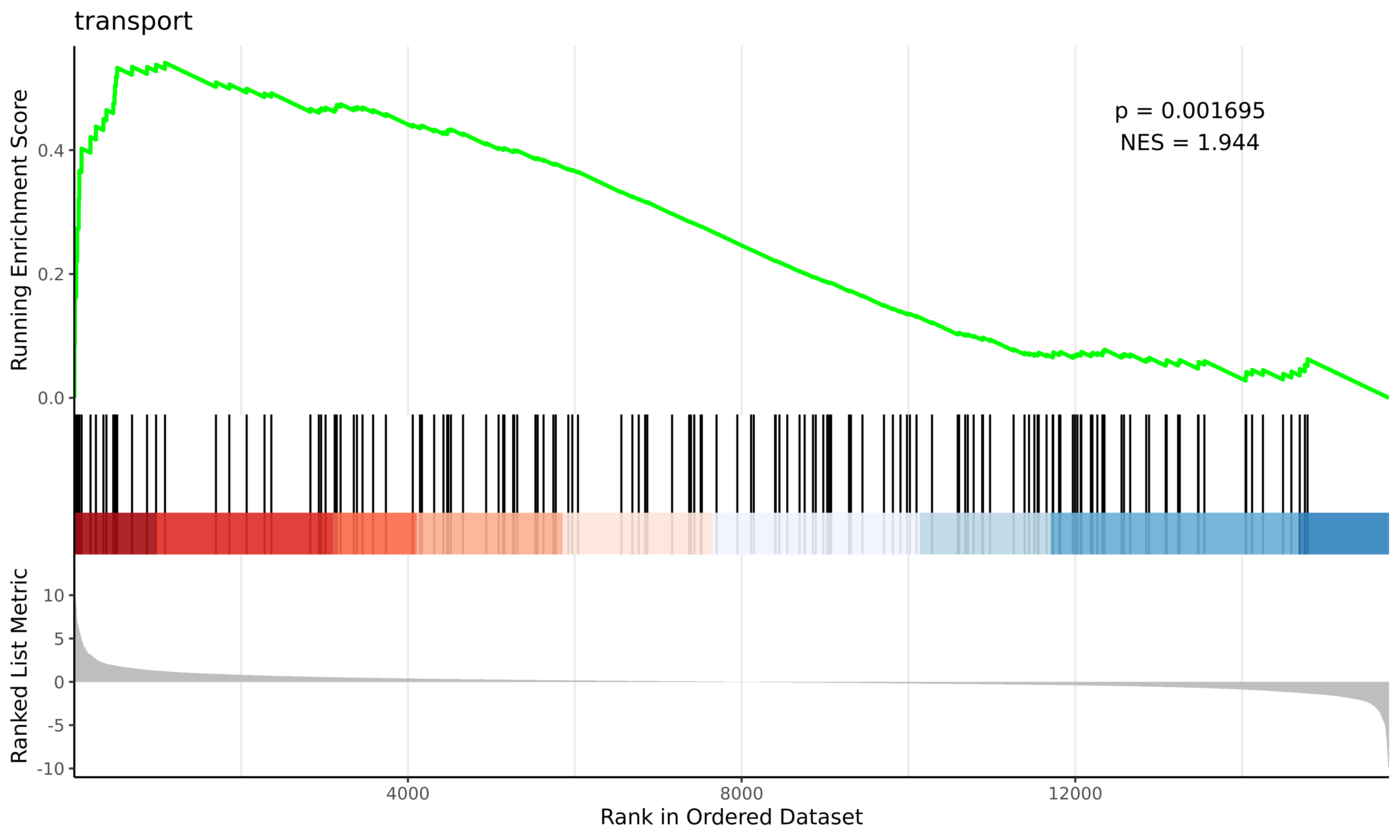

Supplement: Supplementary file 1 [file biology-14-01589-s001.zip › Supplementary Materials S9- GSEenrichment/HYS_vs_LYS_Biological_Process_GO_0006810.gseaplot.png]

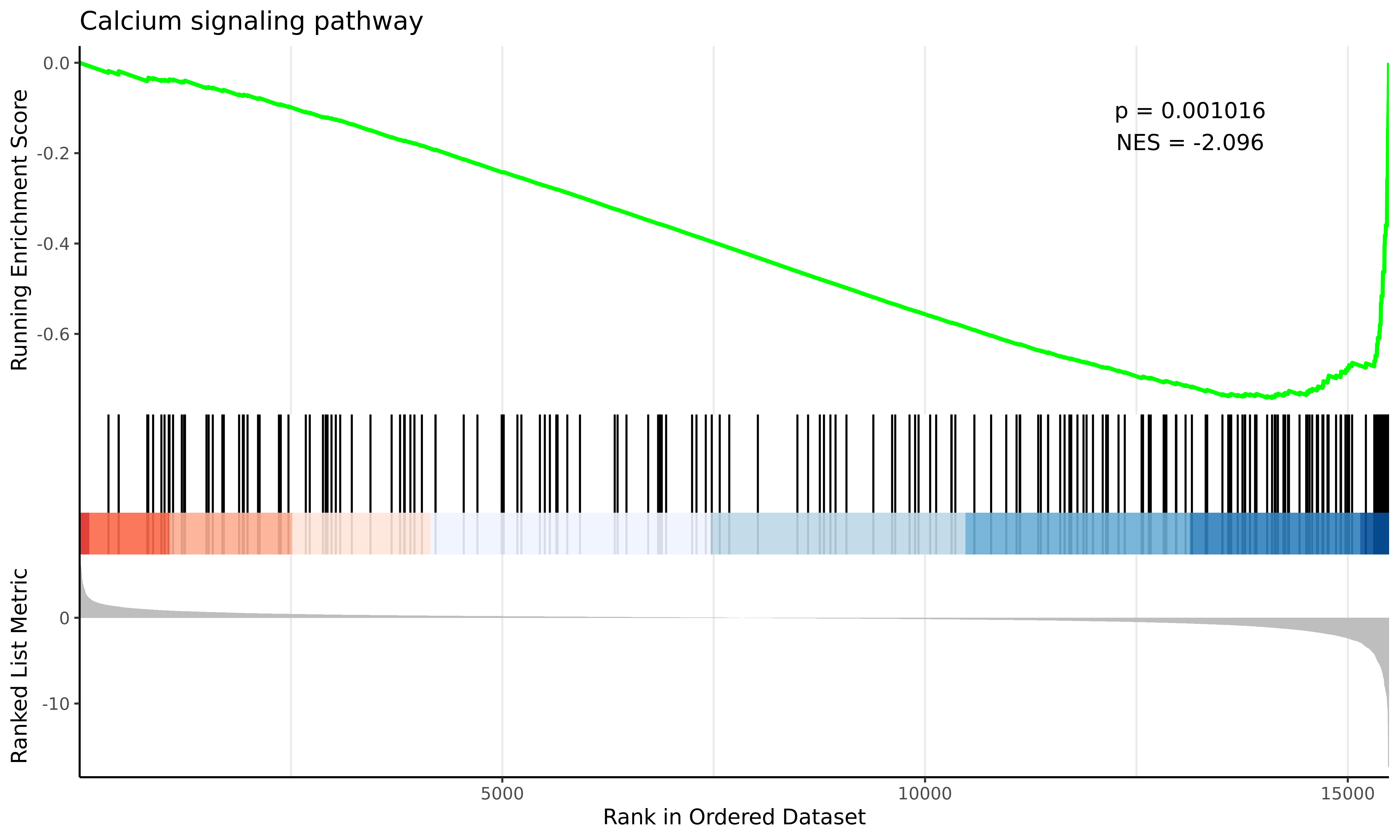

Supplement: Supplementary file 1 [file biology-14-01589-s001.zip › Supplementary Materials S9- GSEenrichment/LYS_vs_CS_KEGG_pathway_ko04020.gseaplot.png]

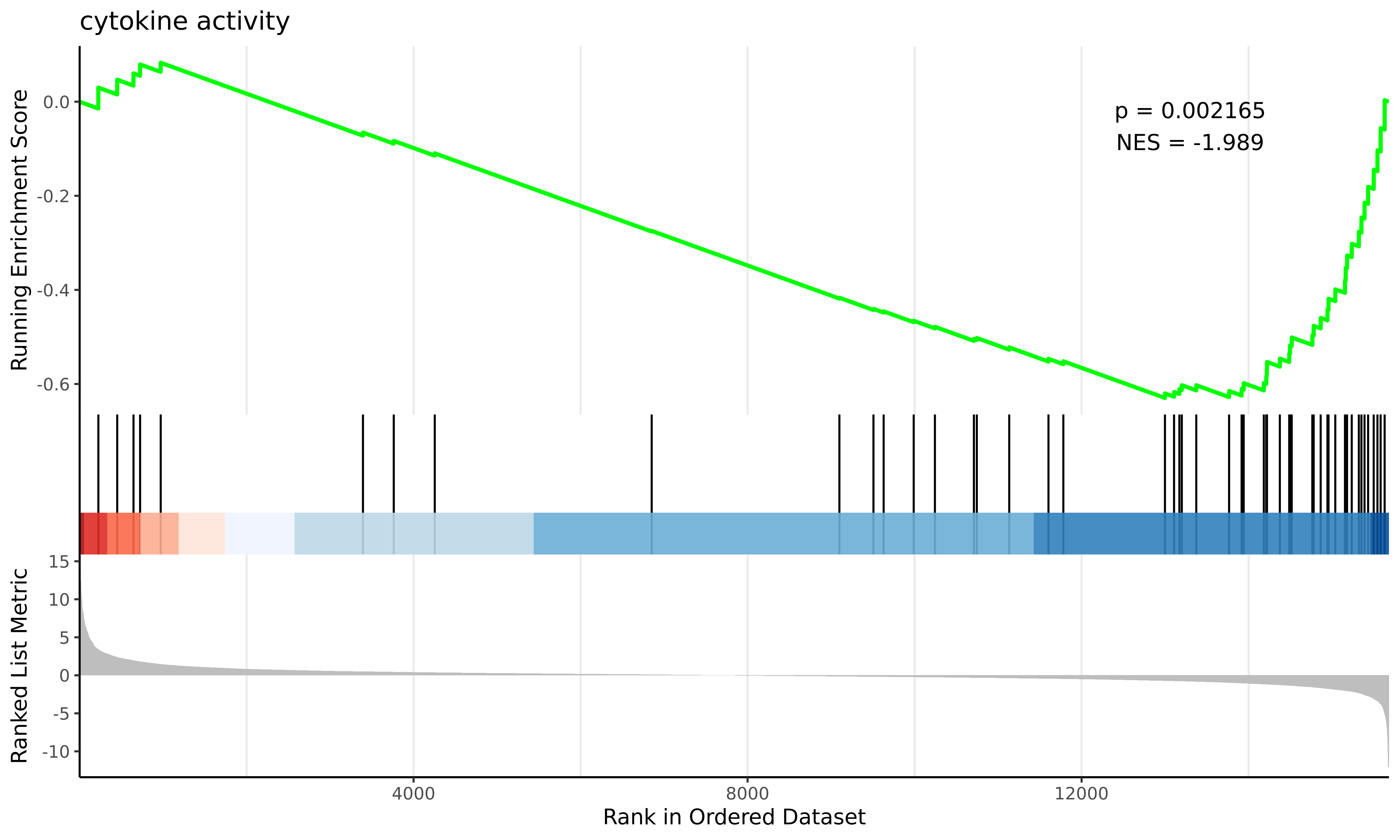

Supplement: Supplementary file 1 [file biology-14-01589-s001.zip › Supplementary Materials S9- GSEenrichment/SMS_vs_CS_Molecular_Function_GO_0005125.gseaplot.png]

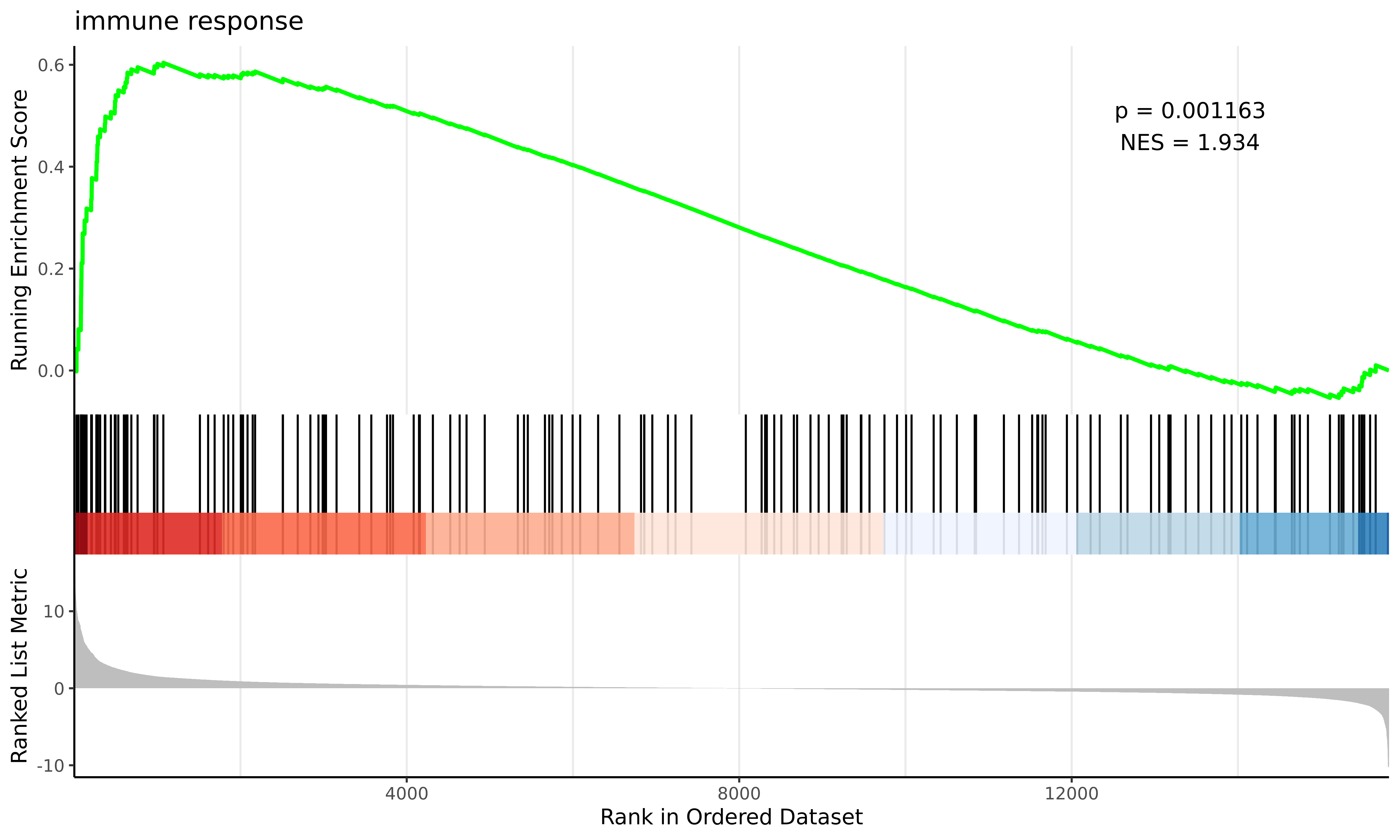

Supplement: Supplementary file 1 [file biology-14-01589-s001.zip › Supplementary Materials S9- GSEenrichment/SMS_vs_LYS_Biological_Process_GO_0006955.gseaplot.png]
